# Minimizing Off‐Target Effects of CRISPR‐Cas9 With Optimized sgRNA: Evaluation of Efficiency and Specificity in the Tumor Protein 53 (TP53) Region

**DOI:** 10.1002/bit.70279

**Published:** 2026-06-28

**Authors:** Ali Mertcan Köse, Monia Ranalli, Drenka Trivanovic, Irina Maslovaric

**Affiliations:** ^1^ Department of Statistics Suleyman Demirel University Isparta Turkey; ^2^ Department of Statistics Sapienza University Rome Italy; ^3^ Institute for Medical Research, University of Belgrade National Institute of the Republic of Serbia Belgrade Serbia

**Keywords:** CRISPR, latent class analysis, levels of off‐target, machine learning, tumor protein 53

## Abstract

CRISPR‐Cas9 is a widely used genetic tool with therapeutic potential in molecular biology. CRISPR‐Cas9 enables precise genome editing by its ability to target specific DNA sequence. After off‐target and on‐target regions are identified, CRISPR‐Cas9 is applied to these regions based on the match between the guide RNA (gRNA) and target DNA sequence. This study points to the off‐target impact of mismatches between the gRNA and target DNA on exon regions of the TP53 gene, which are involved in regulating multiple genes and cellular functions. Off‐target positions are typically evaluated using scoring methods. In this study, we have used latent class analysis to reveal subclasses of off‐target positions. Thus, we have created the levels of off‐target positions and evaluated the effects of mismatching positions within these classes using machine learning classifiers. The results revealed that mismatching positions could be categorized into three levels: low, middle, and high off‐target positions. We have improved a computational framework to minimize off‐target effects and to identify the PAM sequences in the gRNA design. Thus, carefully designed gRNAs will ensure that desired genetic edits are performed and target variants are achieved. This work will avail the future research aimed at optimizing genome editing by customizing CRISPR‐Cas9 to target specific protospacer DNA through gRNA.

## Graphical abstract

Clustered Regularly Interspaced Short Palindromic Repeats (CRISPR) – Cas9‐based genome editing has emerged as a widely used tool across various disciplines, ranging from molecular biology to gene therapy. This revolutionary technology, which enables precise gene editing, represents a significant advancement in biotechnology, opening new frontiers for research and therapeutic applications. This study focuses especially on off‐target positions. In our approach, we encoded the matching between DNA and gRNA using multi‐encoding (4×4 = 16), where combinations of two bases at the same position constitute a variable. To the best of our knowledge, research utilizing Latent Class Analysis in the TP53 region has not been conducted within biological sciences. This study introduces a novel approach for determining off‐target positions using a combination of new encoding systems and statistical methods. Furthermore, off‐target positions are predicted by using machine learning classifiers.

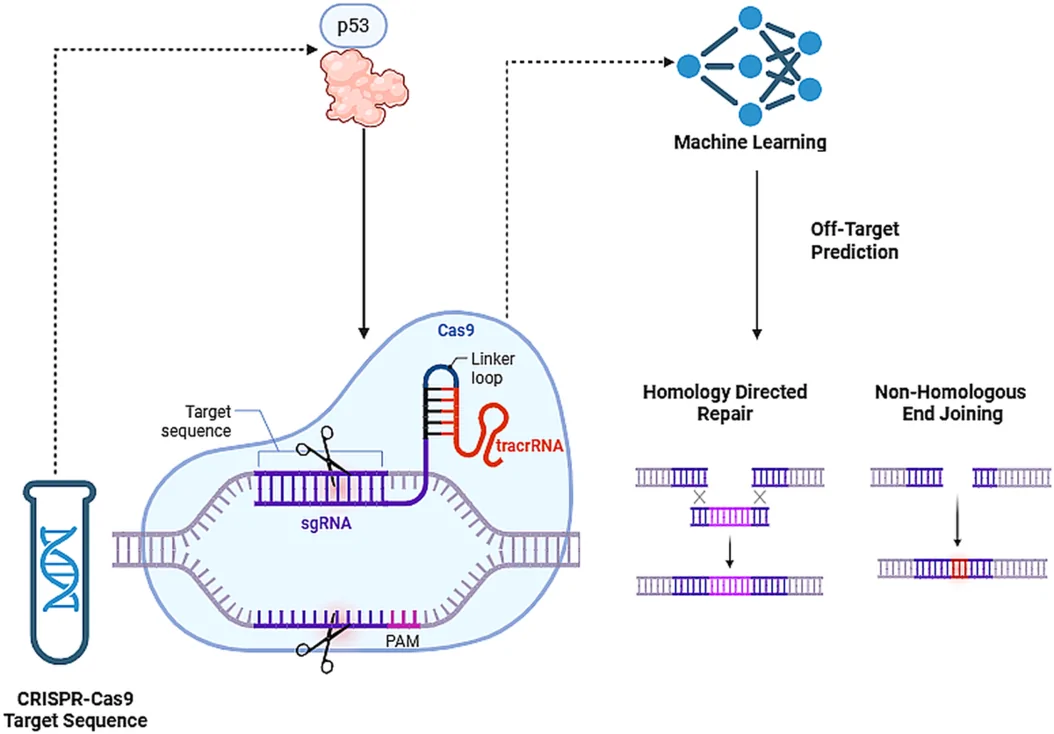

## 1 Introduction

Characterizing genetic diseases with accuracy at the genome‐wide level means, for biologists, comprehensive understanding of all genetic variations across the entire genome to diagnose, manage and understand disease mechanism. The rapid development of genome sequencing technology and bioinformatics enables the detailed description of genetic diseases across different species(Liang et al. 2016). Recently, Clustered Regularly Interspaced Short Palindromic Repeats (CRISPR) – Cas systems, have been used frequently in the field of biology. They emerged as one of the three most potentials major nuclease‐based genome editing tools (Li et al. 2020) that enables the desired modification of various eucaryotic cell genomes through RNA‐guided (gRNA) cleavage of target DNA sequences (Y. Chen and Zhang 2018; Ishino et al. 2018; J. H. Zhang et al. 2016). In this system, Cas9 endonuclease derived from *Streptococcus pyogenes (S.pyogenes)*, and gRNA containing a 20‐nucleotide target recognition sequence homologous to target DNA sequence form a non‐covalent Cas9 ribonucleoprotein bond that cuts a specific DNA target sequence, within the whole genome. Essential for this target DNA sequence cleavage by Cas nuclease is a 3–4 nucleotide long protospacer adjacent motif (PAM) (Chuai et al. 2017; Jiang and Doudna 2017; Liang et al. 2016; Rose et al. 2020). Cas9 endonuclease recognizes a PAM sequence, usually NGG or NAG (N is any nucleobase), found generally downstream from the cut site and adjacent to target DNA sequence (Y. Chen and Zhang 2018; Vora et al. 2022). Thus, Cas9‐induced DNA double‐stranded breaks activate highly efficient DNA repair mechanism through CRISPR‐based gene editing processes. This includes either introducing indels (insertion/deletion) into the DNA donor template via the “Homology Directed Repair (HDR)” method or error‐prone “Non‐Homology End Joining (NHEJ)” method, both of which modify the DNA sequence resulting in targeted integration or gene disruptions, respectively. Researchers can achieve high gene‐cutting precision by combining Cas9 endonuclease enzyme with gRNA carrying a recognition sequence complementary to target DNA sequence (Riesenberg et al. 2022). The ease and high efficiency with which Cas9 site‐specific endonuclease is targeting a specific DNA sequence provides the possibility for multiple modifications (Y. Chen and Zhang 2018; Kang et al. 2020). These DNA repair pathyways rely on precise and efficient CRISPR‐Cas9 mediated gene editing, targeting the desired location. To achieve the outmost of CRISPR‐Cas9 system, the selection of optimal target site and the design of complementary gRNA are crucial steps in a CRISPR‐Cas9 experiment (Wilson et al. 2018). Therefore, detecting both off‐target and on‐target mutations is the most important, as these mutations have a direct impact on the efficiency and reliability of gene editing procedures (Zischewski et al. 2017). Nucleotides can be modified in both off‐target and on‐target regions using knock‐out and knock‐in methods. Although validated gRNA databases are available for various genomes, these libraries are generally designed for knock‐out applications and may not be suitable for certain research purposes, making the custom design of gRNA often necessary (Wilson et al. 2018). In particular, off‐target knock‐out operations are performed on certain transcriptions at the off‐target location, where they are applied by inserting/deleting nucleotides. Herein, off‐target positions are determined by the mismatch between the gRNA and DNA target sequence (Chuai et al. 2017; Leibowitz et al. 2021). Therefore, it is essential to design gRNA libraries that maximize on‐target effects, while minimizing off‐target effects (Chuai et al. 2017; Kuan et al. 2017; Listgarten et al. 2018).

The focus of this study is to develop a model that optimizes specificity and efficiency for all genomic target DNA regions, enabling the detection of viral structures in genome‐wide analysis of genetic diseases through off‐target prediction. There are numerous estimation methods based on mismatching positions, such as CFD, MIT, MIT website score, Cropit, and CCTop Score, that are used to potentially determine off‐target scores (Haeussler et al. 2016). In the literature, CFD and MIT scores are two of the most widely used methods among the numerous estimation methods (Wilson et al. 2018). Considering the number of mismatches and the average distance between them, higher MIT scores indicate less off‐target impact, so guidelines above 50 are recommended (Doench et al. 2016; Haeussler et al. 2016; Hsu et al. 2013). In contrast to the MIT score, the CFD score is an algorithm that provides more accurate off‐target specificity predictions for the CRISPR‐Cas9 system. Unlike the MIT score, the CFD score can assign probability values to each mismatch combination based on the position within the gRNA and the type of mismatch relative to the nucleotide (Fortin et al. 2022). It can be said that the probability of cleavage is higher in regions where the CFD score is above 0.023, and off‐target sites are present (Haeussler et al. 2016). According to Haeussler et al. (2016), the CFD score has the highest accuracy rate of 91%. On the other hand, MIT score 87%, Cropit Score 81%, CCTop score 77% and MIT website score 73% respectively (Haeussler et al. 2016).

It can be stated that computational tools have significantly contributed to the design of optimal multi‐gene editing systems, and it has mutually benefited the optimal CRISPR Cas9 systems (Peng et al. 2021). Although some computational methods are used to determine optimal target sequence for cutting efficiency and to identify potential off‐target editing sites with clusters of gRNAs, we aim to propose a different statistical approach by using latent class analysis (LCA) to suggest an optimal gRNA panel. In this context, LCA is used to aid in determining two or more class levels in a defined population group for a more accurate interpretation and classification of activity scores. The location of off‐target sites can be predicted more precisely by combining machine learning and deep learning approaches with LCA. This will pave the way for estimating multi‐class models with higher precision instead of two‐level class models. Then, it will be possible to systematically test the specific patterns of the mismatch effect and make the minimum possible nucleotide change at each position by using the off‐target classes with LCA. In this study, the editing of the tumor protein 53 (TP53) gene was achieved by prioritizing the off‐target cutting regions of the selected gRNA. In response to stress signals, the p53 protein acts as a transcription factor, which regulates the cell cycle, DNA repair, cell apoptosis, autophagy, and metabolism. The TP53 gene is located on the short arm of chromosome 17 (17p13.1) and is one of the most investigated tumor suppressor genes (Wang et al. 2023). It has been shown that p53 deficiency allows the survival of genomic rearranged cells (Cullot et al. 2023). Additionally, the cutting efficiency of multiple selected gRNA was examined to compare the cutting regions with other edits and to better quantify the differences in cutting efficiency. In the light of this study, the data were first encoded in a multi‐categorical format, and then the levels of off‐target positions were determined using LCA. Subsequently, mismatch positions were predicted at the off‐target levels using both machine learning and deep learning. As a result, this application allowed us to observe the gRNA's effect on the target sequence.

## 2 Methods

### 2.1 Materials and Methods

#### 2.1.1 Data Sources

In the application, analyses were performed using the raw dataset for Homo sapiens tumor protein p53 (TP53), RefSeqGene (LRG_321) on chromosome 17 (https://www.ncbi.nlm.nih.gov/nuccore/NG_017013.2?from=5001&to=24149&report=fasta). The dataset consists of nine variables: gRNA, off‐target sequence, mismatch position, mismatch number, MIT off‐target score, chromosome, start‐end point, strand, and location region. There are 947 possible location regions and 23 endonucleases, including gRNA and target sequence. These variables were transformed into encoded numerical features (V1–V23 mismatch position, mismatch count, and strand) and used as input to the models. The target variable corresponds to the off‐target class levels, representing the categorical outcome of the classification task.

#### 2.1.2 Data Pre‐Processing and Encoding

The off‐target positions consist of 23 endonucleases based on the matching between gRNA and the target sequence. The combination of two bases from the same region is used to create a variable, as the DNA sequence consists of four bases: A/C/G/T. This results in 4×4 = 16 possible combinations for these four bases. These possible mismatching features between the gRNA and target sequence are expressed using multi‐categorical and one‐hot coding labels.

#### 2.1.3 Multi Categorical Coding

In order to perform better analysis on ordinal and nominal measurement levels, variables need to be categorically encoded and further distinguished (Raschka and Mirjalili 2019). The mismatching positions between gRNA and DNA target sequence are encoded using binary coding, where matches are marked as “0” and mismatches are marked as “1.” However, binary coding rules out other mismatched situations and it may present a disadvantage in the analysis. Although binary coding generally provides better performance, multi‐categorical coding minimizes noise in the loss function during classification. Thus, multi‐categorical encoding eliminates classification problems within the framework of risk maximization in machine learning algorithms and provides an advantage (Jia and Zhang 2023; Liu et al. 2022). Multi‐categorical encoding, which refers to the transformation of high‐cardinality categorical variables into numerical representations, can also significantly reduce the computational burden in traditional machine learning models (Zhu et al. 2024).

The mismatched positions between the gRNA and DNA target sequence in the dataset were encoded separately into 16 possible cases, while the matching regions between the gRNA and DNA target sequence were excluded from the coding. Thus, the mismatched positions were categorized into 13 categories, while the matching regions were shown as “1” in Table 1.

**Table 1:** Multi‐categorical coding.

gRNA	Target sequence	Matching
A	A	1
C	A	2
G	A	3
T	A	4
C	C	1
A	C	5
G	C	6
T	C	7
G	G	1
A	G	8
C	G	9
T	G	10
T	T	1
A	T	11
C	T	12
G	T	13

#### 2.1.4 One Hot Coding

**Figure 1:**
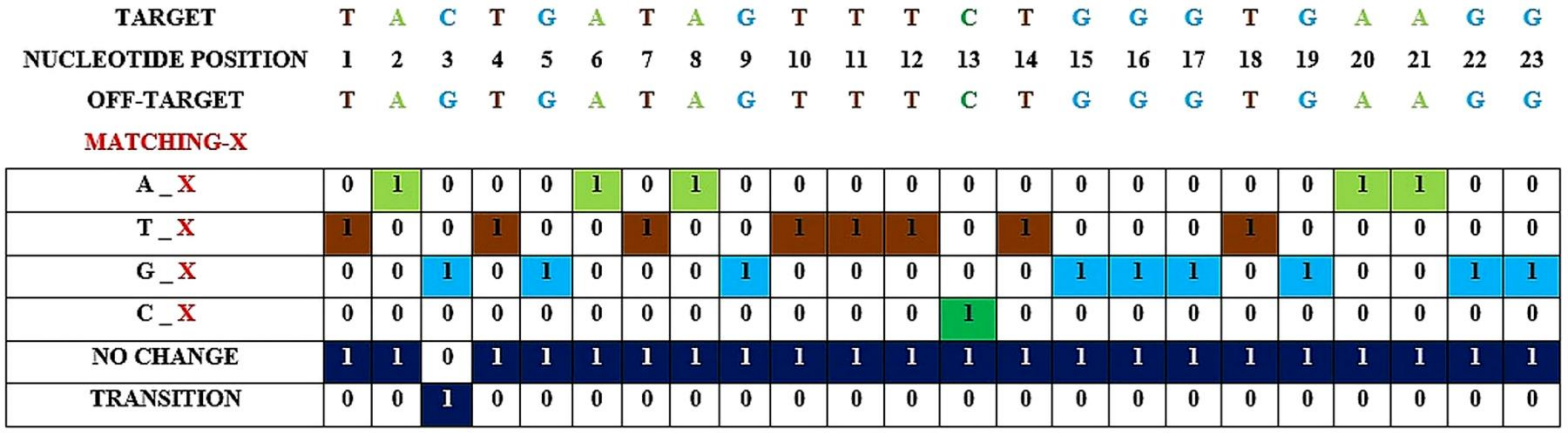
One‐hot encoding for DNA sequence adopted from (Dhanjal et al. 2020).

The one‐hot coding refers to taking a single categorical variable and transforming it into multiple variables, each of which can take on the value 0/1. At most one of these is expressed as “hot” or “one.” The advantage of one‐hot coding can binary categorical inputs to better classify distances or similarities between variables in many algorithms (Yu et al. 2022). The disadvantage is that as the dimensions increase, the model complexity and computational load also increase (Lu 2020). The one‐hot encoding is applied to nucleotide bases as A/C/T/G values, where each base is converted into a vector of length 4. Herein, only one value corresponding to one of the four bases is coded as 1 (“hot”), while the other bases are coded as 0 (Trivedi et al. 2020). Dhanjal et al.(2020) elaborately show the one‐hot encoding through off‐target sequences in Figure 1 (Dhanjal et al. 2020). Multi‐categorical encodings are a more efficient approach to train machine learning classifiers rather than one‐hot encoding. Thereby, one‐hot encoding is a more suitable approach for deep networks in the context of data input structures (Aurélien 2019), and it has been shown that one‐hot encoding is theoretically sufficient for neural networks, as it can reproduce the effects of other encoding schemes through learned transformations, given sufficient data (Zhu et al. 2024).

### 2.2 Latent Class Analysis

The latent class model explains the relationship between observed variables by assuming that each observation unit belongs to one of several latent classes based on the structure of the data population. The latent class model generally consists of two parameters: The first is the relative size of each latent class, and the second is the probability that each observed value belongs to a particular class. There is also an assumption that each latent class has some specific class parameters.

If the latent class model is theoretically defined here, let X= $(X_{1},\ldots ,X_{M}$ *)* represent *M* discrete variables measuring latent variables, where each $X_{m}$ variable can take possible values from 1 to *r*_*m*_. The latent class membership variable is denoted as *C* = 1, 2, …, L. Here, when *Ι(x* = *k)* is represented as an indicator function, it takes the value of 1 if *x* = *k* is 1, and 0 otherwise. If class membership were observed, the joint probability formula, which provides the probability of any observation unit belonging to Class 1 and the corresponding values **x** = g$(x_{1},\ldots ,x_{M}$), is as follows:

$$P\left(X=x,C=l\right)=\gamma_{l}\prod_{m=1}^{M}\prod_{k=1}^{r_{m}}{\pi_{\mathrm{mk}|l}}^{I(X_{m}=k)}.$$

Here,$\;\gamma_{l}$ = P(C = *l*) represents the probability of belonging to latent class *l*, and $\pi_{\mathrm{mk}|l}$ = P ($X_{m}$ = k|C=l) represents the probability of *m* being associated with *k* when considering latent class membership. Therefore, the marginal probability value for a specific response model **x** = $(x_{1},\ldots ,x_{M}$) without considering latent class membership, is as follows:

$$P\left(X=x\right)=\sum_{l=1}^{L}\gamma_{l}\prod_{m=1}^{M}\prod_{k=1}^{r_{m}}{\pi_{\mathrm{mk}|l}}^{I(X_{m}=k)}.$$

There is an assumption of local independence, so that the variables within each class are assumed to be uncorrelated with each other. This assumption is one of the keys of the latent class model that allows us to make inferences about the latent class variable (Chung 2008).

#### 2.2.1 Model Selection

##### 2.2.1.1 Lo–Mendel–Rubin Test (LMRT)

The *k*‐class model is not asymptotically distributed in the latent models, unlike the likelihood ratio model for (k−1) classes. Therefore, the comparison of the two models cannot be evaluated using standard chi‐square difference values. Lo (2001) developed the Vuong–Lo–Mendel–Rubin likelihood test by extending the reference distribution for likelihood ratio within the context of an approximate mixed model, building on the work of Vuong et al.(1989) (Q. Chen et al. 2017; Lo 2001; Vuong 1989). The statistics, defined here as the modified Lo–Mendel–Rubin likelihood ratio test, are formulated as follows:

$${\mathrm{LR}}_{\mathrm{adjusted}}=\frac{\mathrm{LR}}{1+{\left[\left(p-q\right)\log\left(n\right)\right]}^{-1}}.$$

##### 2.2.1.2 Model Selection Criteria

Model selection criteria, such as the Akaike Information Criterion (AIC), Bayesian Information Criterion (BIC), and Consistent Akaike Information Criterion (CAIC), provide frameworks for evaluation models based on their likelihood and complexity. The AIC proposed by Akaike (1973) is a metric used for selecting models estimated from data (Akaike 1973). Due to the overestimation problem associated with AIC, the BIC was proposed by Schwarz (1978) for selecting models estimated from data (Schwarz 1978). CAIC is the derivative of AIC. CAIC was proposed by Bozdogan (1987). It includes a penalty score for models with larger several parameters, adjusted by the sample size *n* (Bozdogan 1987). The following formulas are expressed as follows:

AIC=−2log(L)+2p,

BIC=−2log(L)+p×log(n),

CAIC=−2log(L)+p×(log(n)+1).

Here *L* represents the likelihood function, *p* denotes the number of parameters and *n* is the number of samples (Acquah 2018; Bozdogan 1987, 2000; Nylund et al. 2007).

##### 2.2.1.3 Entropy

The entropy value is an index, which is used to measure the uncertainty of classification. If the posterior probabilities are very similar between classes, it increases the uncertainty of classification. The entropy value ranges from 0 to 1, and it is widely used as a model selection criterion to indicate the level of separation between classes. A higher normalized entropy value represents a better fit for the model. An entropy value higher than 0.60 suggests that the latent class model is quite clearly discriminatory (Asparouhov and Muthén 2014; Qiu 2022; Tein et al. 2013). It is formulated as follows:

$$E=1+\frac{1}{N\log K}\;\sum_{c=1}^{K}\sum_{i=1}^{N}P\left(u|x_{i}\right)\log P\left(u_{c}|x_{i}\right).$$

Here, *K* is the number of classes, *N* is the total number of samples, $x_{i}$ is each observed value, $u_{c}$ represents the observed variables in class *c*, and $P\left(u_{c}|x_{i}\right)$ represents the parameters that estimate the probability of an individual with data $x_{i}$ being in class *c* (Asparouhov and Muthén 2014; Granado 2015; Qiu 2022; Tein et al. 2013).

### 2.3 Machine Learning

The main purpose of machine learning is to analyze data using statistical methods based on datasets. It involves a series of methods that can automatically detect patterns in data and then use these patterns to predict future outcomes (Murphy 2012). In these methods, computers are programmed to optimize performance criteria using data or experience. The model is defined by certain parameters, learning, etc., which is carried out by computer programs to optimize these parameters using training data or past experiences (Alpaydın 2010). In this study, to ensure the validity of class levels obtained from LCA on the CRISPR dataset and to evaluate the effectiveness of scoring (matching) features and chromatin features in CRISPR, the research model was designed and implemented using machine learning algorithms. When class labels are obtained from differential levels by using LCA, the process is resumed through multi‐label classification. Thus, the effects of multi‐label classification are accurately estimated in related classes (Murphy 2012). The classification methods are listed as follows: Logistic Regression (LR) (Efron and Hastie 2016), Linear Discriminant Analysis (LDA) (Alpaydın 2010), K‐Near Neighbors (KNN) (Murphy 2012), Classification Regression Tree (CART) (Murphy 2012), Naïve Bayes (NB) (Mohammed et al. 2017), Support Vector Machine (SVM) (Vasques 2024), Xgboost (Bentéjac et al. 2021), Adaboost (Bishop 2006), Random Forest (RF) (Murphy 2012), Neural Networks (NN) And Convolutional Neural Networks (CNN) (Vasques 2024).

#### 2.3.1 Performance Evaluation Metrics

Performance of any classifier needs to be tested with some metric, to evaluate the predictive model result and the quality of the algorithm for each model, five metrics were considered as classification Accuracy (ACC), Precision, Recall, F1 Score, Calibiration and Receiver Operating Characteristic (ROC) curves. The possible outcomes with respect to this classification is frequently defined in machine learning as true positive (TP), false positive (FP), true negative (TN) and false negative (FN) (Bailly et al. 2022; Q. Cao et al. 2023; Javed et al. 2026; Ozcift and Gulten 2011). The following formulas are expressed as follows:

$$\mathrm{ACC}=\frac{\mathrm{TP}+\mathrm{TN}}{\mathrm{TP}+\mathrm{FP}+\mathrm{TN}+\mathrm{FN}},\;$$

$$\mathrm{Precision}=\frac{\mathrm{TP}}{\mathrm{TP}+\mathrm{FP}},\;$$

$$\mathrm{Recall}=\frac{\mathrm{TP}}{\mathrm{TP}+\mathrm{FN}},\;$$

$$F1\;\mathrm{Score}=\frac{2\times \mathrm{Precision}\times \mathrm{Recall}}{\mathrm{Precision}+\mathrm{Recall}},\;$$

$$\mathrm{AUC}=\frac{1}{2}\left(\frac{\mathrm{TP}}{\mathrm{TP}+\mathrm{FN}}+\frac{\mathrm{TN}}{\mathrm{TN}+\mathrm{FP}}\right),\;$$

$$\mathrm{PR}-\mathrm{AUC}=\int_{0}^{1}\mathrm{Precision}\left(r\right)dr\;\mathrm{where}\;r=\mathrm{recall}.$$

### 2.4 Data Analysis

After preprocessing and encoding the data, the analyses were conducted using latent class and machine learning models. First, latent class models were tested using the LMR test, and the number of classes was determined based on AIC, BIC, CAIC, and entropy. Subsequently, the positions were predicted using machine learning algorithms. The best model was selected based on Accuracy (ACC) and ROC curves. For these analyses, software programs such as R studio (2024.04.2) for LCA and Python (3.10) for machine learning were utilized.

## 3 Results

We used packages with “poLCA” and “ggplot2” in R. In Python, we utilized the following packages: “pandas,” “numpy,” “matplotlib,” “sklearn.datasets,” “sklearn.model_selection,” “sklearn.metrics,” “sklearn.linear_model,” “sklearn.tree,” “sklearn.neighbors,” “sklearn.discriminant_analysis,” “sklearn.naïve_bayes,” “sklearn.svm,” “sklearn.ensemble,” “sklearn.model_selection,” “sklearn.preprocessing,” “imlearn.over_sampling,” “tensorflow,” “tensorflow.keras” and “tensorflow.keras.callbacks.” Algorithm hyperparameters are optimized by the grid search method and early stopping. After the dataset had been split into both train and test datasets, we employed oversampling and regularization methods to address overfitting in the model.

### 3.1 Matching and Mismatching Positions Characteristics

The characteristics of matching and mismatching positions between gRNA and target sequence in the TP53 region are presented in Table 2 for each nucleotide base. The number of non‐matches is 0.1% (*n* = 1), the one‐mismatch count is 2.4% (*n* = 23), the two‐mismatch count is 28.3% (*n* = 268), and the three‐mismatch count is 69.2%(655) in the sequence. While the positive strand is 51.7% (*n* = 490), the negative strand is 48.3% (*n* = 465). Structure of chromotin type floowing as: chrom1 7.9% (*n* = 75), chrom2 8.3% (*n* = 79), chrom4 5.9% (*n* = 56), chrom5 5.4% (*n* = 51), chrom6 5.2% (*n* = 49), chrom7 4.9% (n = 46), chrom8 4.8% (*n* = 45), chrom9 3.9% (*n* = 37), chrom10 5.3% (*n* = 50), chrom11 5.5% (*n* = 52), chrom12 4.4% (*n* = 42), chrom13 3.7% (*n* = 35), chrom14 3.1% (*n* = 29), chrom15 2.1% (*n* = 20), chrom16 4.0% (*n* = 38), chrom17 3.3% (*n* = 31), chrom18 1.8% (*n* = 17), chrom19 2.7% (*n* = 26), chrom20 2.9% (*n* = 27), chrom21 1.4% (*n* = 13), chrom22 2.6% (*n* = 25), chrom23 4.4% (*n* = 42), chromX 0.4% (*n* = 4) respectively.

**Table 2:** Descriptive characteristics of positions.

	Match f (%)	Mismatch f (%)					
Variables	Counts	C‐A/A‐C	G‐A/A‐G	T‐A/A‐T	G‐C/C‐G	T‐C/C‐T	T‐G/G‐T
V1	682 (72.0)	65 (6.9)	28 (2.9)	41 (4.4)	39 (4.1)	45 (4.8)	47 (4.9)
V2	740 (78.1)	17 (1.8)	33 (3.4)	66 (6.9)	40 (4.2)	17 (1.8)	34 (3.4)
V3	788 (83.2)	31 (3.3)	16 (1.6)	31 (3.3)	30 (3.2)	20 (2.1)	31 (3.2)
V4	777 (82.0)	6 (0.6)	35 (3.7)	29 (3.1)	20 (2.1)	25 (2.6)	55 (5.8)
V5	750 (79.2)	41 (4.4)	70 (7.4)	15 (1.6)	46 (4.9)	13 (1.4)	12 (1.3)
V6	807 (85.2)	17 (1.8)	29 (3.0)	34 (3.6)	12 (1.2)	41 (4.3)	7 (0.7)
V7	718 (75.8)	24 (2.5)	93 (9.8)	61 (6.4)	9 (1.0)	—	42 (4.4)
V8	592 (62.5)	33 (3.5)	125 (13.2)	—	99 (10.5)	39 (4.1)	59 (6.2)
V9	707 (74.7)	6 (1.1)	50 (5.3)	47 (4.9)	44 (4.6)	51 (5.3)	42 (4.4)
V10	748 (79.0)	18 (1.9)	31 (3.2)	36 (3.8)	35 (3.7)	59 (6.2)	20 (2.2)
V11	662 (69.9)	131 (13.9)	27 (2.8)	30 (3.1)	31 (3.2)	43 (4.5)	23 (2.5)
V12	649 (68.5)	67 (7.1)	80 (8.4)	19 (2.0)	56 (5.9)	37 (3.9)	39 (4.2)
V13	748 (79.0)	72 (7.6)	36 (3.8)	30 (3.2)	12 (1.3)	47 (5.0)	2 (0.2)
V14	756 (79.8)	84 (8.9)	15 (1.5)	39 (4.2)	10 (1.0)	36 (3.8)	7 (0.7)
V15	750 (79.2)	84 (8.8)	15 (1.6)	21 (2.2)	32 (3.4)	40 (4.2)	5 (0.5)
V16	675 (71.3)	54 (5.7)	29 (3.1)	92 (9.7)	23 (2.5)	18 (1.9)	56 (5.9)
V17	631 (66.6)	32 (3.4)	38 (4.1)	45 (4.7)	24 (2.6)	103 (10.9)	74 (7.8)
V18	690 (72.9)	24 (2.5)	35 (3.7)	19 (2.0)	36 (3.8)	93 (9.8)	50 (5.3)
V19	689 (72.8)	62 (6.6)	20 (2.2)	42 (4.4)	65 (6.9)	42 (4.5)	27 (2.8)
V20	741 (78.2)	44 (4.6)	19 (2.0)	62 (6.5)	13 (1.4)	27 (2.8)	41 (4.4)
V21	255 (26.9)	113 (12.0)	129 (13.6)	144 (15.2)	83 (8.8)	106 (11.2)	117 (12.3)
V22	454 (47.9)	—	493 (52.1)	—	—	—	—
V23	947 (100)	—	—	—	—	—	—

### 3.2 Latent Class Analysis

After pre‐processing and coding the data, the off‐target positions were determined based on the mismatched bases between the gRNA and target sequence. Afterwards, the latent class model was employed to ascertain the number of levels in off‐target positions. The LMR test results were statistically significant (< 0.001) for each latent class in Table 3. Therefore, Information Criteria (AIC, BIC, CAIC) were considered for model selection to fit each latent class. According to AIC, we didn't specify the optimal model. Although AIC is widely used in model selection for predictive models, it is known to favor more complex models and may not always be optimal for determining the true underlying class structure (Chakrabarti and Ghosh 2011). Therefore, the BIC, which is recommended in LCA for penalizing model complexity more rigorously and generally seeks to identify the least complex and best interpretable model, was given greater weight. Furthermore, the LMR test and model interpretability were also taken into account in selecting the final three class solution. Consequently, the model was estimated with three classes, which was the optimal solution based on BIC, CAIC, and entropy. Due to the latent class model's entropy value greater than 0.60, the identified latent class model was found highly distinctive (Asparouhov and Muthén 2014; Qiu 2022; Tein et al. 2013).

**Table 3:** Results of latent class analysis for multi‐categorical observed variables.

Class	Parameter	Log Likelihood	AIC	BIC	CAIC	LMR Test	Entropy
**2 class**	449	−21753.8	44405.6	46584.7	47033.7	< 0.001	0.953
**3 class**	674	−20798.8	42945.6	**46216.7**	**46880.7**	**< 0.001**	**0.981**
**4 class**	899	−20330.9	42459.7	46822.8	47721.8	< 0.001	0.977
**5 class**	1124	−19815.4	41878.8	47333.9	48457.9	< 0.001	0.977
**6 class**	1349	−19170	41037.9	47585.0	48934.1	< 0.001	0.989

**Figure 2:**
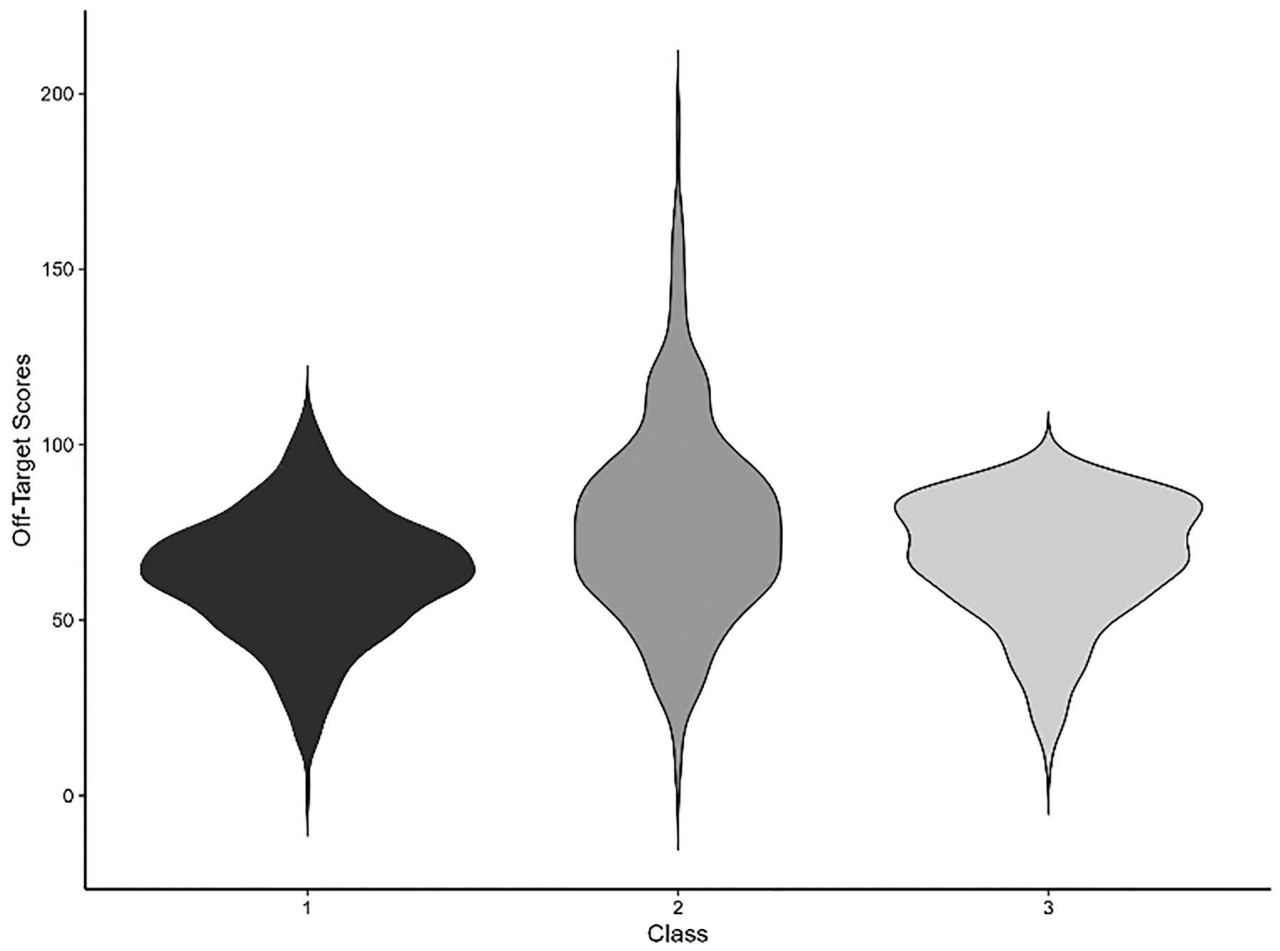
Off‐Target scores for each class.

Off‐Target scores are examined for each class in Figure 2. According to comparisons of class results, there have been statistically significant differences between off‐target class levels and MIT off‐target scores (Kruskal–Wallis *H*: 58.749; *p* < 0.001). The highest scores are obtained in the second class (77.8 ± 28.0), then the third (67.1 ± 17.6), and the first class (62.5 ± 17.6), respectively. We can label classes as high for the second class, middle for the third class, and low for the first class.

### 3.3 Machine Learning

A three‐level off‐target model was obtained using latent class models as a result of the mismatch between the gRNA and the target sequence. Subsequently, the three off‐target levels were predicted through machine learning algorithms based on mismatch positions, mismatch count, chromosome, and strand. All prediction metrics related to models are presented in Table 4. CNN has given the highest ACCs and other performance indicators, such as the precision, recall, and F1 measures, for both train and test datasets.

**Table 4:** The Machine Learning classifiers' results.

		Train (70%)				Test (30%)			
Model (Nested cross‐validation)	Hyperparameters	ACC	Precision	Recall	F1 score	ACC	Precision	Recall	F1 Score
Logistic regression (LR)	Solver: liblinear Regularization (C): 10	0.758	0.760	0.756	0.757	0.674	0.677	0.672	0.674
Linear discriminant analysis (LDA)	Default	0.754	0.756	0.751	0.753	0.667	0.677	0.665	0.670
K‐near neighbors (KNN)	Number of neighbors (k): 7	0.749	0.760	0.731	0.740	0.642	0.712	0.609	0.626
Classification regression tree (CART)	Maximum depth: 10 Minimum samples split: 10	0.873	0.884	0.867	0.873	0.818	0.833	0.819	0.833
Naïve Bayes (NB)	Default	0.701	0.700	0.712	0.702	0.663	0.662	0.679	0.665
Support vector machine (SVM)	Kernel: RBF Regularization parameter (C): 10 Gamma: auto	0.814	0.819	0.805	0.810	0.716	0.740	0.705	0.715
Xgboost	Number of estimators: 100 Learning rate: 0.01 Maximum depth: 4	0.959	0.963	0.957	0.960	0.912	0.924	0.908	0.914
Adaboost	Number of estimators: 100 Learning rate: 0.01 Maximum depth: 4	0.908	0.916	0.905	0.910	0.800	0.828	0.799	0.810
Random forest (RF)	Number of trees: 100 Maximum depth: 5	0.944	0.952	0.939	0.945	0.856	0.872	0.854	0.862
Neural network (NN)	Activation: ReLU Optimizer: Adam Learning rate: 0.001 Batch size: 32 Number of epochs: 50 Drop rate: 0.5 L2 regularization: 0.001	0.996	0.996	0.996	0.996	0.950	0.956	0.955	0.952
**Convolutional neural network (CNN)**	**Activation: ReLU** **Optimizer: Adam** **Learning rate:0.001** **Batch size: 32** **Number of epochs: 50** **Drop rate:0.5** **L2 regularization:0.001**	**0.999**	**0.999**	**0.999**	**9.999**	**0.960**	**0.959**	**0.964**	**0.961**

*Note:* Bold values indicate the best‐performing model in each column.

**Figure 3:**
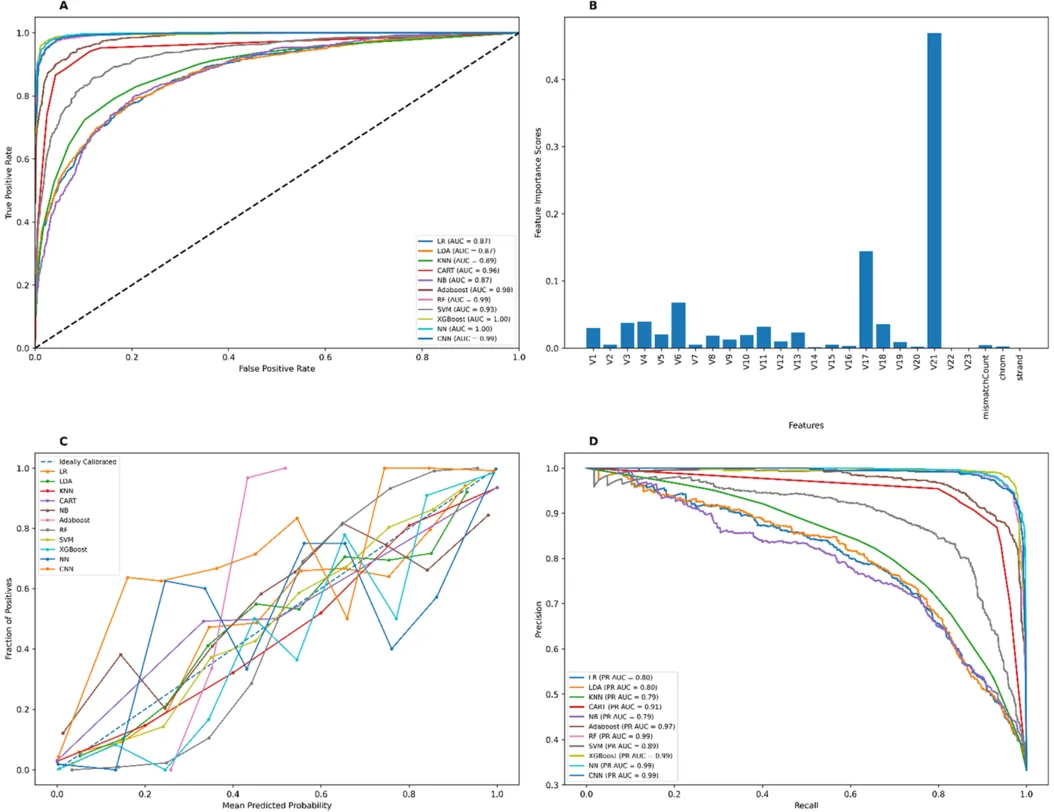
(A) Combined ROC curve for multiple classifiers. (B) Feature importance for the XGBoost algorithm. (C) Calibration Curves. (D) Precision‐Recall Area Under the Curves (PR‐AUC).

The ROC Curve for the model estimated from all algorithms is shown in Figure 3. CNN and Xgboost have the highest area under the curve (AUC) values of 1.00, indicating that these models perfectly discriminate between classes. According to the model estimated using the XGBoost algorithm, the most important features are highlighted in Figure 3. Especially, these features include mismatching positions (21st position, 17th position, 6th position, 4th position, and 3rd position). Herein, the 17th position shows a strong positive effect on off‐target prediction across all datasets. We also found that 3rd, 4th, and 6th positions contribute positively to the TP53 gene dataset. On the other hand, the finding that the 21st position (PAM's nucleotide) may accelerate the transitions between gRNA and DNA, facilitating off‐target cleavage, made it the most important variable in this study.

## 4 Discussion

In this study, the effect of off‐target regions in the CRISPR Cas9 system is investigated using LCA in the TP53 gene. Before the analysis, the data were encoded in a format suitable for machine learning classifiers to better understand the mismatches between gRNA and target sequences. This encoding process identified potential mismatches in the 23‐nucleotide long segment, including the PAM site within the gRNA sequence. Each mismatch case was handled separately using multi‐categorical coding as the result of 16 possible scenarios. However, the matching regions between the gRNA and the target sequence were excluded from this coding, and the analysis focused solely on the mismatches. This coding process created 13 different categories of mismatches. Matching regions were coded as “1” and included in the analyses. A multi‐categorical method was used for general analyses. On the other hand, the data was processed using the one‐hot encoding method for deep learning algorithms (NN and CNN), so the algorithms could analyze the mismatches more effectively. The CRISPR Cas9 system's effects were examined on the off‐target regions in the TP53 gene using both encoding approaches.

LCA was first used to determine the subclasses of off‐target positions. Thus, the level of target sites was determined by the number and location of mismatches between the gRNA and target sequences. While machine learning models are generally estimated as on/off‐target for CRISPR applications in the literature, this research estimated machine learning models across more than two off‐target levels using latent class models based on off‐target positions. After each latent class model was tested with the LMR test, models were selected as the three latent classes using information criteria from the significant models. One of the most significant challenges in the use of CRISPR gene editing is off‐target DNA cleavage, where the molecular shears alter the wrong region of the target genome, leading to non‐specific, unwanted, unexpected, and even undesirable changes in the genome. When these errors occur, the system generates genetic mutations instead of correcting them. To minimize off‐target effects, off‐target effects were examined at three levels using LCA to optimize sgRNA. Thus, off‐target genome editing undermines and reduces the therapeutic potential of CRISPR‐Cas9. The three classes of off‐target effects can be significantly evaluated to minimize undesirable DNA damage and adverse events such as immune responses and cytotoxicity (Mengstie et al. 2024). Then, machine learning algorithms were used to estimate the positions of the off‐target levels. Off‐target positions were estimated using Logistic Regression, Linear Discriminant Analysis, K‐Nearest Neighbors, Regression Trees, Naïve Bayes, Support Vector Machines, Xgboost, Adaboost, Random Forest, Neural Networks, and Convolutional Neural Networks classifiers. It was found that the XGBoost algorithm with multi‐categorical encoding achieved the highest accuracy in both the training and test datasets. According to the analysis results, off‐target levels contribute positively to the increase in accuracy in neural networks (train: 99.6%, test: 95%) and convolutional neural network applications (train: 99.9%, test: 96%), which were implemented using the one‐hot encoding method. Deep learning classifiers were supported by other performance ratios, including precision, recall, and F1 for both the train and test dataset. Furthermore, deep networks, boosting algorithms, decision trees, and support vector machines were notably discriminated from traditional classifiers such as logistic regression, linear discriminant analysis, K‐nearest neighbors, and naïve Bayes. The model was also estimated by using the XGBoost algorithm. Important features included mismatching positions (21st position, 17th position, 6th position, 4th position and 3rd position). First, experimental studies in the literature have examined the location of off‐target effects in regions proximal to the PAM. Second, the binding of gRNA and DNA in regions distal to the PAM has also been investigated in relation to off‐target effects. In contrast, mismatches at more upstream positions do not significantly affect the stability of the gRNA–DNA duplex and therefore have minimal impact on the cleavage process (J. S. Chen et al. 2017; Modrzejewski et al. 2020). These findings are consistent with studies showing that nucleotides at positions 8–12 adjacent to the PAM are critical determinants of Cas9 specificity. Moreover, recent studies by Vora et al. (2023) and Zhang et al. (2024) have demonstrated that positions further away from the PAM can also provide valuable information regarding off‐target activity (Vora et al. 2023; G. Zhang et al. 2024).

There are many studies on off‐target prediction using both machine learning and deep learning in the literature. While Kose et al. (2024) used hybrid models with machine learning and LCA models, other studies obtained accuracy using traditional machine learning algorithms. These studies are shown in Table 5.

**Table 5:** A summary of studies applying machine learning methods for off‐target in CRISPR‐Cas9 by Sherkatghanad et al. (2023).

Study	Models	Prediction metric/results
(Vora et al. (2022))	RF	ACC: 0.970
(S. A. Chen and Tran (2018))	LR	ACC: 0.940
(Aktaş et al. (2019))	NNET	ACC: 0.960
(Z. R. Zhang and Jiang (2022))	SVM	ACC: 0.982
(Dhanjal et al. (2020))	Xgboost	ACC: 0.914
(Kose et al. (2024))	Xgboost	ACC: 1.000
(Trivedi et al. (2020))	SVM	AUC: 0.980
(Chuai et al. (2018))	CNN (DeepCRISPR)	AUC: 0.804
(Toufikuzzaman et al. (2024))	CNN (DIPOFF)	ACC: 0.997
(Sun et al. (2024))	CNN (CRISPR‐M)	ACC: 0.968
(M. Cao et al. (2025))	CNN (CRISPR‐Net)	AUC: 0.852
(Abadi et al. (2017))	RF	AUC: 0.960
(Peng et al. (2018))	RF	AUC: 0.990
(Lazzarotto et al. (2020))	RF	AUC: 0.995
(Listgarten et al. (2018))	Boosted Regression Trees	AUC: 0.980
(Doench et al. (2016))	SVM + LR	AUC: 0.800
(S. Zhang et al. (2019))	Adaboost	AUC: 0.938

*Note:* Bold values indicate the best‐performing model in each column.

Machine learning and deep learning techniques hold great potential in genome editing studies, it provides high accuracy and classification rates in off‐target prediction. Especially, algorithms demonstrate superior performance in both ACC and AUC metrics, such as random forest, support vector machines, and convolutional neural networks. Toufikuzzaman et al. (2024), Vora et al. (2022), and Z.R. Zhang and Jiang (2022) indicate significant progress in this field; they have found ACC of over 97%. In AUC metrics, Lazzarotto et al. (2020) and Peng et al. (2018) achieved success rates above 99%. Konstantakos et al. (2022) express that getting the most out of gRNA is actually getting the most out of a complex interplay of factors such as sequence composition, secondary structure, experimental conditions, and numerous other factors, yet unknown (Konstantakos et al. 2022). Machine learning models can capture this complex interplay of parameters and produce CRISPR gene‐editing models. However, Konstantakos et al. (2022) point to shortcomings of existing models, such as sensitivity to data heterogeneity, an insufficient training dataset, and an inadequate ability to produce general gRNA design rules. Hence, further efforts are required to improve in‐silico gRNA design for high on‐target (efficiency) activity and to reduce off‐target (specificity) effects (Konstantakos et al. 2022). The ability to determine off‐target cleavage activity of the CRISPR‐Cas9 system, which is presented in a recent investigation as a strategy for curing HIV‐1. It is crucial for the clinical progression of gene editing (Atkins et al. 2021). Because the position determines what happens as a result. It also follows from our study that the predictive ability of each tool will vary depending on its intended use. If the model is applied to data that are similar to its training dataset, it will likely achieve an acceptable performance. In general, Konstantakos et al. (2022) show that learning‐based tools can adopt the model to particular data, despite the limited biological knowledge of the CRISPR‐Cas9 mechanism. Unlike Kose et al. (2024), who examined classes in three regions (Intron, Intergenic, and Exon), LCA and machine learning were applied solely to the exon region in this study. One of the recent studies by Wu et al. (2025) presents how LCA analysis and machine learning could be put to use in clinical substyping prediction and differentiation in suspected neurosyphilis patients. On the other hand, the absence of a gold standard diagnostic criterion in diseases like neurosyphilis, multiple sclerosis, and others, due to their heterogeneous clinical manifestations and variable treatment responses, makes the LCA classification of patients into homogenous, clinically meaningful latent subtypes imperative in facilitating early diagnosis and treatment of disease. According to Wu et al. (2025), findings validated the biological relevance of clinical characteristics classified into subgroups by LCA (Wu et al. 2025). The present study is based on computational modeling; however, related aspects of the approach have been validated using an external dataset in our previous work (Kose et al. 2024). Nevertheless, the present work is still limited by the absence of independent experimental validation. Moreover, a convolutional neural network model was incorporated into this study. This study demonstrates that LCA and machine learning applications performed only on exons give better results in determining off‐target levels. It also shows that the one‐hot encoding method provides more meaningful results in predicting outcomes using neural networks and convolutional neural network methods. In the future direction, we plan to hybridize our procedure with combined models.

## 5 Conclusion

Consequently, to minimize off‐target effects, the gRNA must exactly match the PAM sequence. The PAM is recognized by the Cas nuclease and serves as a signal for binding and cutting the target sequence. Especially, the PAM sequence is required to initiate cutting at the target sequence in some CRISPR systems, such as Cas9. If the suitable PAM is not found in the target sequence by the gRNA, Cas9 or a similar enzyme will not be able to cut the DNA, and genetic editing will not occur. Selecting a suitable PAM sequence can reduce the risk of undesirable off‐target effects and increase the accuracy of genetic editing processes. Therefore, addressing the right PAM sequence plays a crucial role in genetic editing studies using the CRISPR Cas system. gRNA allows the CRISPR Cas system to direct the target sequence. This allows genetic editing to occur directly at the desired site, as gRNA can be highly specific to the target sequence. Thus, unwanted off‐target effects are reduced, increasing the precision of the genetic editing process. This also enables specific changes to be made in specific regions of certain genes. gRNA design offers a wide range of applications for editing various genes or targeted genetic regions. Therefore, gRNA is a crucial component in genetic editing studies using Cas systems. Carefully designed gRNAs ensure that the desired genetic edits are made, and targeted variants are achieved. These findings offer valuable insights into the complex interactions between CRISPR gRNA and genetic sequences. It has been suggested that a new strategy for development is to address the challenges caused by genomic composition and the significant differences between the gRNA and DNA target regions. This study contributes to potential future research aimed at optimizing genome editing by customizing CRISPR Cas9 to target specific protospacer DNA.

## Conflicts of Interest

The authors declare no conflicts of interest.

## Supporting information

Supporting File

## Data Availability

The data that support the findings of the study are available from the corresponding author upon reasonable request.
