# Supplementary material for: Minimizing Off‐Target Effects of CRISPR‐Cas9 With Optimized sgRNA: Evaluation of Efficiency and Specificity in the Tumor Protein 53 (TP53) Region
Source: Biotechnol Bioeng. 2026 Jun 28;123(10):2677–89. doi: 10.1002/bit.70279 (PMC13576857; doi:10.1002/bit.70279)
Supplement: Supplementary file 1 [file BIT-123-2677-s001.pdf]

Biotechnology Journal
Systems & Synthetic Biology · Nanobiotech · Medicine

Research Article

Minimizing Off-Target Effects of CRISPR-Cas9 with Optimized sgRNA: Evaluation of Efficiency and Specificity in the Tumor Protein 53 (TP53) Region

**Submission ID**	519b0479-4547-4121-947b-3b7ca6469d35
**Submission Version**	Initial Submission
**PDF Generation**	02 Mar 2026 06:00:03 EST by Atypon ReX

Authors

Dr. Ali Mertcan Köse
Corresponding Author
Submitting Author

CRediT
Conceptualization, Investigation, Funding acquisition, Writing - original draft, Methodology, Validation, Writing - review & editing, Software, Formal analysis, Project administration, Resources, Supervision, Data curation

Affiliations
- Department of Statistics, Suleyman Demirel University, Isparta, Turkey

Dr. Monia Ranalli

ORCiD
https://orcid.org/0000-0001-7193-8803

CRediT
Investigation, Validation, Methodology, Software, Formal analysis, Writing - review & editing, Supervision, Conceptualization

Affiliations
- Department of Statistics, Sapienza University, Rome, Italy

Dr. Drenka Trivanovic

CRediT
Investigation, Writing - review & editing, Validation, Methodology, Supervision, Conceptualization

Affiliations
- Institute for Medical Research, University of Belgrade, Belgrade, Serbia

Dr. Irina Maslovaric

ORCiD
https://orcid.org/0000-0003-0892-5617

CRediT
Writing - review & editing, Validation, Investigation, Conceptualization, Supervision, Methodology

Affiliations
- Institute for Medical Research, University of Belgrade, Belgrade, Serbia

Files for peer review

All files submitted by the author for peer review are listed below. Files that could not be converted to PDF are indicated; reviewers are able to access them online.

Name	Type of File	Size	Page
Anonymous_Minimizing Off-Target Effects.docx	Main Document - MS Word	502.7 KB	Page 4
Figures.docx	Figure	320.0 KB	Page 30
Tables.docx	Table	42.9 KB	Page 32
Highlights-.docx	Novelty Statement	13.0 KB	Page 37
Graphical Abstract.png	Graphical Abstract Image	914.2 KB	Page 38

Minimizing Off-Target Effects of CRISPR-Cas9 with Optimized sgRNA: Evaluation of Efficiency and Specificity in the Tumor Protein 53 (TP53) Region

Ali Mertcan Köse[a*], Monia Ranalli[b], Drenka Trivanovic[c], Irina Maslovaric[c]

[a]*Department of Statistics, Suleyman Demirel University, Isparta, Turkey*

[b]*Department of Statistics, Sapienza University, Rome, Italy*

[c]*Institute for Medical Research, University of Belgrade, Belgrade, Serbia*

Funding Details: No

Conflict of interest: The authors have no conflict of interest to disclosure

Data availability statement: The datasets involved in this study are publicly available from the following links:

The raw data: https://www.ncbi.nlm.nih.gov/nuccore/NG_017013.2?from=5001&to=24149&report=fasta

The application data: https://github.com/mertcank1/CRISPR_Cas9_Off_Target_Prediction_TP53.

Data availability statement: The datasets involved in this study are publicly available from the following links:

Code is available at https://github.com/mertcank1/CRISPR_Cas9_Off_Target_Prediction_TP53.

Acknowledgements

Part of this work was completed when the corresponding author visited the School of Statistical Sciences at Sapienza University during the Erasmus+ Program. We would like to thank Dr. Alessandra Recchia and Dr. Daniela Benati for their critical comments on this study. Also, the author's collaboration in this work was provided by Cost Action (CA21113)-Genome Editing to Treat Human Diseases. This study was presented at the 15th Scientific Meeting Classification and Data Analysis Group (CLADAG 2025) by the corresponding author.

*Corresponding Author: Ali Mertcan Köse, alikose@sdu.edu.tr, Suleyman Demirel University, Faculty of Engineering and Natural Sciences, Department of Statistics PO Box 32260, Isparta, Turkey.

Abstract

CRISPR-Cas9 is a widely used genetic tool with therapeutic potential in molecular biology. CRISPR-Cas9 enables precise genome editing by its ability to target specific DNA sequence. After Off-Target and On-Target regions are identified, CRISPR-Cas9 is applied to these regions based on the match between the guide RNA (gRNA) and target DNA sequence. This study points to the Off-Target impact of mismatches between the gRNA and target DNA on exon regions of the TP53 gene which are involved in regulating multiple genes and cellular functions. Off-target positions are typically evaluated using scoring methods. In this study, we have used latent class analysis to reveal subclasses of Off-Target positions. Thus, we have created levels of Off-Target positions and evaluated the effects of mismatching positions within these classes using machine learning classifiers. The results revealed that mismatching positions could be categorized into three levels: low, middle, and high Off-Target positions. We have minimized Off-Target effects and identified the PAM sequences in the gRNA design. Carefully designed gRNAs ensure that desired genetic edits are performed, and targeted variants are achieved. This work will avail future research aimed at optimizing genome editing by customizing CRISPR-Cas9 to target protospacer DNA through gRNA.

Keywords:Latent class analysis, machine learning, CRISPR, levels of Off-Target, Tumor Protein 53

1. Introduction

Accurately characterizing genetic diseases at the genome-wide level is a critical function for biologists. The rapid development of genome sequencing technology and bioinformatics enables the detailed description of genetic diseases across different species [1]. Recently, CRISPR (Clustered Regularly Interspaced Short Palindromic Repeats) –Cas systems, have been used frequently in the field of biology. They emerged as one of most potential out of the three major nuclease-based genome editing tools [2] that enables the desired modification of various eucaryotic cell genomes through RNA-guided (gRNA) cleavage of target DNA sequences [3], [4], [5]. In this system, Cas9 endonuclease derived from *Streptococcus pyogenes (S.pyogenes)*, and gRNA containing a 20-nucleotide target recognition sequence homologous to target DNA sequence form a non-covalent Cas9 ribonucleoprotein bond that cuts a specific DNA target sequence, within the whole genome. Essential for this target DNA sequence cleavage by Cas nuclease is a 3-4 nucleotide long protospacer adjacent motif (PAM)[1], [6], [7], [8]. Cas9 endonuclease recognizes a PAM sequence, usually NGG or NAG (N is any nucleobase), found generally downstream from the cut site and adjacent to target DNA sequence [4], [9]. Thus, Cas9-induced DNA double-stranded breaks activate highly efficient DNA repair mechanism through CRISPR-based gene editing processes, either by introducing indels (insertion/deletion) into the DNA donor template via the "Homology Directed Repair (HDR)" method or by using the "Non-Homology End Joining (NHEJ)" method, both of which modify the DNA sequence resulting in targeted integration or gene disruptions, respectively. Researchers can achieve high gene-cutting precision by combining Cas9 with gRNA carrying a recognition sequence complementary to target DNA sequence [10]. The ease with which Cas9 targeting a specific DNA sequence allows its high efficiency as a site-specific endonuclease and enables the possibility of multiple modifications[4], [11]. All of these approaches require precise and efficient targeting of the CRISPR-Cas9 system to the desired location. The success of an experiment using the CRISPR-Cas9 system depends on correctly identifying the optimal target site and designing the complementary gRNA [12]. Therefore, detecting both Off-Target and On-Target mutations is the most important, as these mutations have a direct impact on the efficiency and reliability of gene editing procedures [13]. Nucleotides can be modified in both Off-Target and On-Target regions using Knock-Out and Knock-In Methods. Although validated gRNA databases are available for various genomes, these libraries are generally designed for Knock-Out applications and may not be suitable for certain research purposes, making the custom design of gRNA often necessary [12].In particular, Knock-

Out operations are performed on certain transcription at the Off-Target location, where they are applied by inserting/deleting nucleotides. Herein, Off-Target positions are determined by the mismatch between the gRNA and target sequence [7], [14]. Therefore, it is essential to design gRNA libraries that maximize On-Target effects, while minimizing Off-Target effects [7], [15], [16].

The focus of this study is to develop a model that optimizes specificity and efficiency for all genomic target DNA regions, enabling the detection of viral structures in genome-wide analysis of genetic diseases through Off-Target prediction. There are numerous estimation methods based on mismatching positions, such as CFD, MIT, MIT website score, Cropit, and CCTop Score, that are used to potentially determine Off-Target Scores [17]. In the literature, CFD and MIT scores are two of the most widely used methods among the numerous estimation methods [12]. Considering the number of mismatches and the average distance between them, higher MIT scores indicate less Off-Target impact, so guidelines above 50 are recommended [17], [18], [19]. In contrast to the MIT score, the CFD score is an algorithm that provides more accurate Off-Target specificity predictions for the CRISPR-Cas9 system. Unlike the MIT score, the CFD score is weighted based on the position and type of mismatch relative to the nucleotide [20]. It can be said that the probability of cleavage is higher in regions where the CFD score is above 0.023 and Off-Target sites are present [17]. According to Haeussler et al.(2016), the CFD score has the highest accuracy rate of 91%. On the other hand; MIT score 87%,, Cropit Score 81%, CCTop score 77% and MIT website score 73% respectively [17].

It can be stated that computational tools have significantly contributed to the design of optimal multi-gene editing systems and it has mutually benefited the optimal CRISPR Cas9 systems [21]. Although some existing computational methods are used to determine on target sequence for cutting efficiency and potential Off-Target editing sites with clusters of gRNAs, we aim to propose a different statistical approach by using latent class analysis to suggest an optimal gRNA panel. In this context, latent class analysis aids in determining two or more class levels for a more accurate interpretation and classification of activity scores. The location of Off-Target sites can be predicted more precisely by combining machine learning and deep learning approaches with latent class analysis. This will pave the way for estimating multi-class models with higher precision instead of two-level class models. Thus, it will be possible to systematically test the mismatch effect and make the minimum possible nucleotide change at each position by using the Off-Target class. In this study, the

editing of the tumor protein 53 (TP53) gene was achieved by prioritizing the Off-Target cutting regions of the selected gRNA. In response to stress signals, p53 protein acts as a transcription factor, which regulates cell cycle, DNA repair, cell apoptosis, autophagy, and metabolism. TP53 gene is located on the short arm of chromosome 17 (17p13.1) and is one of the most investigated tumor suppressor genes [22], and it has been shown that p53 deficiency allows the survival of genomic rearranged cells [23]. Additionally, the cutting efficiency of multiple selected gRNA was examined to compare the cutting regions with other edits and to better quantify the differences in cutting efficiency. In the light of this study, the data were first encoded in a multi-categorical format, and then the levels of Off-Target positions were determined using Latent Class Analysis. Subsequently, mismatch positions were predicted at the Off-Target levels using both machine learning and deep learning. As a result, this application allowed us to observe the gRNA's effect on the target sequence.

2. Methods

2.1.Materials and Methods

2.1.1. Data Sources

In the application, analyses were performed using the raw dataset for Homo sapiens tumor protein p53 (TP53), RefSeqGene (LRG_321) on chromosome 17 (https://www.ncbi.nlm.nih.gov/nuccore/NG_017013.2?from=5001&to=24149&report=fasta). The dataset consists of nine variables: gRNA, off-target sequence, mismatch position, mismatch number, MIT off-target score, chromosome, start-end point, strand, and location region. There are 947 possible location regions and 23 endonucleases, including gRNA and target sequence.

2.1.2. Data Pre-processing and Encoding

The Off-Target positions consist of 23 endonucleases based on matching between gRNA and the target sequence. The combination of two bases from the same region is used to create a variable as the DNA sequence

consists of four bases: A/C/G/T. This results in 4×4 = 16 possible combinations for these four bases. These possible mismatching features between the gRNA and target sequence are expressed using multi-categorical and one-hot coding labels.

2.1.3. Multi Categorical Coding

In order to perform better analysis on ordinal and nominal measurement levels, variables need to be categorically encoded and further distinguished [24]. The mismatching positions between gRNA and target sequence are encoded using binary coding, where matches are marked as '0' and mismatches are marked as '1'. However, binary coding rules out other mismatched situations and it may present a disadvantage in the analysis. Although binary coding generally provides better performance, multi-categorical coding minimizes noise in the loss function during classification. Thus multi-categorical encoding eliminates classification problems within the framework of risk maximization in machine learning algorithms and provides an advantage [25], [26].

The mismatched positions between the gRNA and target sequence in the dataset were encoded separately into 16 possible cases, while the matching regions between the gRNA and target sequence were excluded from the coding. Thus, the mismatched positions were categorized into 13 categories, while the matching regions were shown as '1' in Table 1.

Table 1. Multi-categorical coding

gRNA	Target Sequence	Matching
A	A	1
C	A	2
G	A	3
T	A	4
C	C	1
A	C	5
G	C	6
T	C	7
G	G	1
A	G	8
C	G	9
T	G	10

T	T	1
A	T	11
C	T	12
G	T	13

2.1.4. One Hot Coding

The One-hot coding refers to taking a single categorical variable and transforming it into multiple variables, each of which can take on the value 0/1. At most one of these is expressed as 'hot' or 'one'. The advantage of one-hot coding can binarize categorical inputs to better classify distances or similarities between variables in many algorithms [27]. The disadvantage is that as the dimensions increase, the model complexity and computational load also increase [28]. The one-hot encoding is applied to nucleotide bases as A/C/T/G values, where each base is converted into a vector of length 4. Herein, only one value corresponding to one of the four bases is coded as 1 ('hot'), while the other bases are coded as 0 [29]. Dhanjal et al. [30] elaborately shows the one-hot encoding through off-target sequences in Figure 1 [30]. Multi-categorical encodings are a more efficient approach to train machine learning classifiers rather than one-hot encoding. Thereby, one-hot encoding is a more suitable approach for deep networks in the context of data input structures [31].

TARGET	T	A	C	T	G	A	T	A	G	T	T	T	C	T	G	G	G	T	G	A	A	G	G
NUCLEOTIDE POSITION	1	2	3	4	5	6	7	8	9	10	11	12	13	14	15	16	17	18	19	20	21	22	23
OFF-TARGET	T	A	G	T	G	A	T	A	G	T	T	T	C	T	G	G	G	T	G	A	A	G	G
MATCHING-X																							
A_X	0	1	0	0	0	1	0	1	0	0	0	0	0	0	0	0	0	0	0	1	1	0	0
T_X	1	0	0	1	0	0	1	0	0	1	1	1	0	1	0	0	0	1	0	0	0	0	0
G_X	0	0	1	0	1	0	0	0	1	0	0	0	0	0	1	1	1	0	1	0	0	1	1
C_X	0	0	0	0	0	0	0	0	0	0	0	0	1	0	0	0	0	0	0	0	0	0	0
NO CHANGE	1	1	0	1	1	1	1	1	1	1	1	1	1	1	1	1	1	1	1	1	1	1	1
TRANSITION	0	0	1	0	0	0	0	0	0	0	0	0	0	0	0	0	0	0	0	0	0	0	0

Figure 1. One-hot encoding for DNA sequence adopted from [30]

2.2.Latent Class Analysis

The latent class model explains the relationship between observed variables by assuming that each observation unit belongs to one of several latent classes based on the structure of the data population. The latent class model generally consists of two parameters: The first is the relative size of each latent class, and the second is the probability that each observed value belongs to a particular class. There is also an assumption that each latent class has some specific class parameters.

If the latent class model is theoretically defined here, let $\boldsymbol{X}= (X_1,\ldots,X_M)$ represent M discrete variables measuring latent variables, where each X_m variable can take possible values from 1 to r_m. The latent class membership variable is denoted as $C = 1, 2, \ldots, L$. Here, when $I(x=k)$ is represented as an indicator function, it takes the value of 1 if $x=k$ is 1, and 0 otherwise. If class membership were observed, the joint probability formula, which provides the probability of any observation unit belonging to Class 1 and the corresponding values $\mathbf{x} = (x_1,\ldots,x_M)$, is as follows:

$$P(X = x, C = l) = \gamma_l \prod_{m=1}^{M} \prod_{k=1}^{r_m} \pi_{mk|l}{}^{I(X_m=\mathrm{k})}$$

Here, γ_l=P(C=l) represents the probability of belonging to latent class l, and $\pi_{mk|l}$ = P (X_m=k|C = l) represents the probability of m being associated with k when considering latent class membership. Therefore, the marginal probability value for a specific response model $\mathbf{x} = (x_1,\ldots,x_M)$ without considering latent class membership, is as follows:

$$P(X = x) = \sum_{l=1}^{L} \gamma_l \prod_{m=1}^{M} \prod_{k=1}^{r_m} \pi_{mk|l}{}^{I(X_m=\mathrm{k})}$$

There is an assumption of local independence, so that the variables within each class are assumed to be uncorrelated with each other. This assumption is the one of the keys the latent class model that allows us to make inferences about the latent class variable [32].

2.2.1. Model Selection

2.2.1.1.Lo-Mendel-Rubin Test (LMRT)

The k-class model is not asymptotically distributed in the latent models, unlike the likelihood ratio model for ($k-1$) classes. Therefore, the comparison of the two models cannot be evaluated using standard chi-square difference values. Lo et al.[33] developed the Vuong-Lo-Mendel-Rubin likelihood test by extending the reference distribution for likelihood ratio within the context of an approximate mixed model, building on the work of Vuong et al. [34] [33], [35]. The statistic, defined here as the modified Lo-Mendel-Rubin likelihood ratio test, is formulated as follows:

$$LR_{adjusted} = \frac{LR}{1 + [(p-q)\log(n)\,]^{-1}}$$

2.2.1.2.Model Selection Criteria

Model selection criteria, such as the Akaike Information Criterion (AIC), Bayesian Information Criterion (BIC), and Consistent Akaike Information Criterion (CAIC), provide frameworks for evaluation models based on their likelihood and complexity. The AIC proposed by Akaike [36] is a metric used for selecting models estimated from data [36]. Due to the overestimation problem associated with AIC, the Bayesian Information Criterion (BIC) was proposed by Schwarz [37] for selecting models estimated from data [37]. CAIC is the derivative of AIC. CAIC was proposed by Bozdogan [38]. It includes a penalty score for models with larger several parameters, adjusted by the sample size *n* [38]. The following formulas are expressed as follows:

$$AIC = -2\log(L) + 2p$$

$$BIC = -2\log(L) + p \times \log(n)$$

$$CAIC = -2\log(L) + p \times (\log(n) + 1)$$

Here L represents the likelihood function, p denotes the number of parameters and n is the number of samples [38], [39], [40], [41].

2.2.1.3. *Entropy*

The entropy value is an index, which is used to measure the uncertainty of classification. If the posterior probabilities are very similar between classes, it increases the uncertainty of classification. The entropy value ranges from 0 to 1 and it is widely used as a model selection criterion to indicate the level of separation between

classes. A higher normalized entropy value represents a better fit for the model. An entropy value higher than 0.60 suggests that the latent class model is quite clearly discriminatory [42], [43], [44]. It is formulated as follows:

$$E=1+\frac{1}{NlogK}\sum_{c=1}^{K}\sum_{i=1}^{N}P(u\,|\,x_i)logP(u_c\,|\,x_i)$$

Here, K is the number of classes, N is the total number of samples, x_i is each observed value, u_c represents the observed variables in class c, and $P(u_c\,|\,x_i)$ represents the parameters that estimate the probability of an individual with data x_i being in class c [42], [43], [44], [45].

2.3.Machine Learning

The main purpose of machine learning is to analyze data using statistical methods based on datasets. It involves a series of methods that can automatically detect patterns in data and then use these patterns to predict future outcomes [46]. In these methods, computers are programmed to optimize performance criteria using data or experience. The model is defined by certain parameters, learning, etc., which is carried out by computer programs to optimize these parameters using training data or past experiences [47]. In this study, to ensure the validity of class levels obtained from latent class analysis on the CRISPR dataset and to evaluate the effectiveness of scoring (matching) features and chromatin features in CRISPR, the research model was designed and implemented using machine learning algorithms. When class labels are obtained from differential levels by using latent class analysis, the process is resumed through multi-label classification. Thus the effects of multi-label classification are accurately estimated in related classes [46]. The classification methods are listed as follows: Logistic Regression [48], Linear Discriminant Analysis [47], K-Near Neighbors [46], Classification Regression Tree [46], Naïve Bayes [49], Support Vector Machine [50], Xgboost [51], Adaboost [52], Random Forest [46], Neural Network And Convolutional Neural Network [50].

2.3.1. Performance Evaluation Metrics

Performance of any classifier needs to be tested with some metric, to evaluate the predictive model result and the quality of the algorithm for each model, five metrics were considered as classification Accuracy (ACC), Precision, Recall, F1 Score and Receiver Operating Characteristic (ROC) curves. The possible outcomes with respect to this classification is frequently defined in machine learning as true positive (TP), false positive (FP), true negative (TN) and false negative (FN) [53], [54], [55]. The following formulas are expressed as follows:

$$ACC=\frac{TP+TN}{TP+FP+TN+FN}$$

$$Precision = \frac{TP}{TP + FP}$$

$$Recall = \frac{TP}{TP + FN}$$

$$F1\ Score = \frac{2 \times Precision\ \times Recall}{Precision + Recall}$$

$$AUC = \frac{1}{2}\left(\frac{TP}{TP + FN} + \frac{TN}{TN + FP}\right)$$

2.4.Data Analysis

After preprocessing and encoding the data, the analyses were conducted using latent class and machine learning models. First, latent class models were tested using the LMR test, and the number of classes was determined based on AIC, BIC, CAIC, and entropy. Subsequently, the positions were predicted using machine learning algorithms. The best model was selected based on Accuracy (ACC) and Receiver Operating Characteristic (ROC) curves. For these analyses, software programs such as R studio (2024.04.2) for LCA and Python (3.10) for machine learning were utilized.

3. Results

We used packages with "poLCA" and "ggplot2"in R. In Python, we utilized the following packages: "pandas", "numpy", "matplotlib", "sklearn.datasets", "sklearn.model_selection", "sklearn.metrics", "sklearn.linear_model", "sklearn.tree", "sklearn.neighbors", "sklearn.discriminant_analysis", "sklearn. naïve_bayes", "sklearn.svm", "sklearn.ensemble", "sklearn.model_selection", "sklearn.preprocessing", "imlearn.over_sampling", "tensorflow", "tensorflow.keras" and "tensorflow.keras.callbacks". Algorithm hyperparameters are optimized by the grid search method and early stopping. Furthermore, we employed oversampling and regularization methods to address overfitting in the model.

3.1.Matching and Mismatching Positions Characteristics

The characteristics of matching and mismatching positions between gRNA and target sequence in the TP53 region are presented in Table 2 for each nucleotide base.

Table 2. Descriptive characteristics of positions

Variables	Frequency	Percent	Variables	Frequency	Percent

		(n=947)	(%)			(n=947)	(%)
V1	match-1	682	72	V14	match-1	756	79.8
	C-A/A-C	65	6.9		C-A/A-C	84	8.9
	G-A/A-G	28	2.9		G-A/A-G	15	1.5
	T-A/A-T	41	4.4		T-A/A-T	39	4.2
	G-C/C-G	39	4.1		G-C/C-G	10	1
	T-C/C-T	45	4.8		T-C/C-T	36	3.8
	T-G/G-T	47	4.9		T-G	7	0.7
V2	match-1	740	78.1	V15	match-1	750	79.2
	C-A/A-C	17	1.8		C-A/A-C	84	8.8
	G-A/A-G	33	3.4		G-A	15	1.6
	T-A/A-T	66	6.9		T-A/A-T	21	2.2
	G-C/C-G	40	4.2		G-C	32	3.4
	T-C/C-T	17	1.8		T-C/C-T	40	4.2
	T-G/G-T	34	3.4		G-T	5	0.5
V3	match-1	788	83.2	V16	match-1	675	71.3
	C-A/A-C	31	3.3		C-A/A-C	54	5.7
	G-A/A-G	16	1.6		G-A/A-G	29	3.1
	T-A/A-T	31	3.3		T-A/A-T	92	9.7
	G-C/C-G	30	3.2		G-C/C-G	23	2.5
	T-C/C-T	20	2.1		T-C/C-T	18	1.9
	T-G/G-T	31	3.2		T-G/G-T	56	5.9
V4	match-1	777	82	V17	match-1	631	66.6
	C-A	6	0.6		C-A/A-C	32	3.4
	G-A/A-G	35	3.7		G-A/A-G	38	4.1
	T-A/A-T	29	3.1		T-A/A-T	45	4.7
	C-G	20	2.1		G-C/C-G	24	2.6
	C-T	25	2.6		T-C/C-T	103	10.9
	T-G/G-T	55	5.8		T-G/G-T	74	7.8
V5	match-1	750	79.2	V18	match-1	690	72.9
	C-A/A-C	41	4.4		A-C	24	2.5
	G-A/A-G	70	7.4		/A-G	35	3.7
	T-A	15	1.6		A-T	19	2
	G-C/C-G	46	4.9		G-C/C-G	36	3.8
	T-C	13	1.4		T-C/C-T	93	9.8
	T-G	12	1.3		T-G/G-T	50	5.3
V6	match-1	807	85.2	V19	match-1	689	72.8
	C-A/A-C	17	1.8		C-A/A-C	62	6.6
	G-A/A-G	29	3		G-A/A-G	20	2.2
	T-A/A-T	34	3.6		T-A/A-T	42	4.4
	G-C/C-G	12	1.2		G-C/C-G	65	6.9
	T-C/C-T	41	4.3		T-C/C-T	42	4.5
	T-G	7	0.7		T-G/G-T	27	2.8
V7	match-1	718	75.8	V20	match-1	741	78.2
	C-A	24	2.5		C-A/A-C	44	4.6
	G-A/A-G	93	9.8		G-A/A-G	19	2
	T-A	61	6.4		T-A/A-T	62	6.5
	C-G	9	1		G-C/C-G	13	1.4
	T-G	42	4.4		T-C/C-T	27	2.8
					T-G/G-T	41	4.4
V8	match-1	592	62.5	V21	match-1	255	26.9
	A-C	33	3.5		C-A/A-C	113	12
	A-G	125	13.2		G-A/A-G	129	13.6
	G-C/C-G	99	10.5		T-A/A-T	144	15.2

	T-C	39	4.1		G-C/C-G	83	8.8
	T-G	59	6.2		T-C/C-T	106	11.2
					T-G/G-T	117	12.3
V9	match-1	707	74.7	V22	match-1	454	47.9
	C-A/A-C	6	1.1		A-G	493	52.1
	G-A/A-G	50	5.3	V23	match-1	947	100
	T-A/A-T	47	4.9	mismatchCount	0	1	0.1
	G-C/C-G	44	4.6		1	23	2.4
	T-C/C-T	51	5.3		2	268	28.3
	T-G/G-T	42	4.4		3	655	69.2
V10	match-1	748	79	Strand	+	490	51.7
	C-A/A-C	18	1.9		-	457	48.3
	G-A/A-G	31	3.2	class	1	401	42.3
	T-A/A-T	36	3.8		2	253	26.7
	G-C/C-G	35	3.7				
	T-C/C-T	59	6.2		3	293	30.9
	T-G/G-T	20	2.2				
V11	match-1	662	69.9	chrom	chrom1	75	7.9
	C-A/A-C	131	13.9		chrom2	79	8.3
	G-A/A-G	27	2.8		chrom3	58	6.1
	T-A/A-T	30	3.1		chrom4	56	5.9
	G-C/C-G	31	3.2		chrom5	51	5.4
	T-C/C-T	43	4.5		chrom6	49	5.2
	T-G/G-T	23	2.5		chrom7	46	4.9
V12	match-1	649	68.5		chrom8	45	4.8
	C-A/A-C	67	7.1		chrom9	37	3.9
	G-A/A-G	80	8.4		chrom10	50	5.3
	T-A/A-T	19	2		chrom11	52	5.5
	G-C/C-G	56	5.9		chrom12	42	4.4
	T-C/C-T	37	3.9		chrom13	35	3.7
	T-G/G-T	39	4.2		chrom14	29	3.1
V13	match-1	748	79		chrom15	20	2.1
	C-A/A-C	72	7.6		chrom16	38	4
	G-A/A-G	36	3.8		chrom17	31	3.3
	T-A	30	3.2		chrom18	17	1.8
	G-C/C-G	12	1.3		chrom19	26	2.7
	T-C	47	5		chrom20	27	2.9
					chrom21	13	1.4
	T-G	2	0.2		chrom22	25	2.6
					chrom23	42	4.4
					chromX	4	0.4

3.2.Latent Class Analysis

After pre-processing and coding the data, the off-target positions were determined based on the mismatched bases between the gRNA and target sequence. Afterwards, the latent class model was employed to ascertain the number of levels in off-target positions. The LMR test results were statistically significant (<0.001) for each latent class

in Table 3. Therefore, Information Criteria (AIC, BIC, CAIC) were considered for model selection to fit each latent class. According to AIC, the fitted model couldn't be selected. However, the model was estimated with three classes, which was the optimal solution based on BIC, CAIC, and entropy. Due to the latent class model's entropy value greater than 0.60, the identified latent class model was found highly distinctive [42], [43], [44].

Table 3. Results of latent class analysis for multi-categorical observed variables

Class	Parameter	Log Likelihood	AIC	BIC	CAIC	LMR Test	Entropy
**2 class**	449	-21753.8	44405.6	46584.7	47033.7	<0.001	0.953
**3 class**	674	-20798.8	42945.6	**46216.7**	**46880.7**	**<0.001**	**0.981**
**4 class**	899	-20330.9	42459.7	46822.8	47721.8	<0.001	0.977
**5 class**	1124	-19815.4	41878.8	47333.9	48457.9	<0.001	0.977
**6 class**	1349	-19170	41037.9	47585.0	48934.1	<0.001	0.989

Off-Target scores are examined for each class in Figure 2. According to comparisons of class results, there have been statistically significant differences between Off-Target class levels and MIT Off-Target Scores (Kruskal Wallis H: 58.749; p<0.001). The highest scores are obtained in the second class (77.8 ± 28.0), then the third (67.1 ± 17.6), and the first class (62.5 ± 17.6) respectively. We can label classes as high for the second class, middle for the third class and low for the first class.

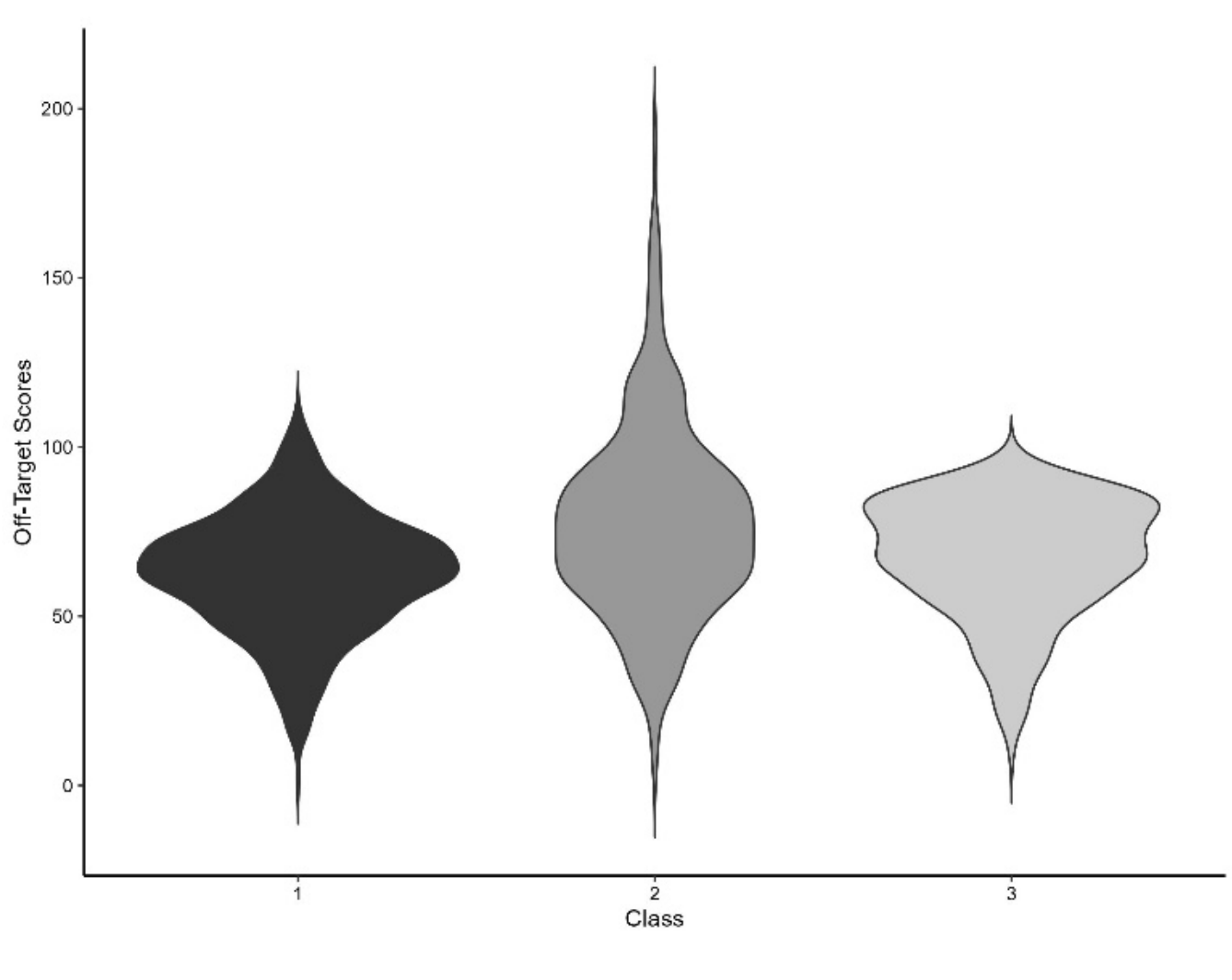

Figure 2. Off-Target scores for each class.

3.3. Machine Learning

A three-level Off-Target model was obtained using latent class models as a result of the mismatch between the gRNA and the target sequence. Subsequently, the three Off-Target levels were predicted through machine learning algorithms based on mismatch positions, mismatch count, chromosome, and strand. All prediction metrics related to models are presented in Table 4. CNN has given the highest ACCs and other performance indicators such as the precision, recall and F1 measures for both train and test datasets.

Table 4. The Machine learning classifiers results

	**Train (70%)**				**Test (30%)**			
**Model (10-fold cross validation)**	**ACC**	**Precision**	**Recall**	**F1 score**	**ACC**	**Precision**	**Recall**	**F1 Score**
Logistic regression (LR)	0.760	0.760	0.760	0.760	0.663	0.664	0.663	0.663
Linear discriminant analysis (LDA)	0.754	0.754	0.754	0.753	0.667	0.669	0.667	0.666
K-near neighbors (KNN)	0.766	0.774	0.766	0.763	0.604	0.645	0.604	0.592
Classification regression tree (CART)	0.872	0.861	0.861	0.861	0.811	0.824	0.821	0.821
Naïve Bayes (NB)	0.701	0.711	0.701	0.701	0.663	0.670	0.663	0.661
Support vector machine (SVM)	0.917	0.920	0.916	0.916	0.772	0.785	0.771	0.770
Xgboost	1.000	1.000	1.000	1.000	0.951	0.955	0.954	0.954
Adaboost	0.784	0.789	0.784	0.786	0.744	0.749	0.744	0.746
Random forest (RF)	0.947	0.934	0.932	0.932	0.854	0.860	0.853	0.854
Neural network (NN)	1.000	1.000	1.000	1.000	0.989	0.980	0.979	0.979
**Convolutional neural network (CNN)**	**1.000**	**1.000**	**1.000**	**1.000**	**1.000**	**1.000**	**1.000**	**1.000**

The ROC Curve for the model estimated from all algorithms is shown in Figure 3. CNN and Xgboost have the highest area under the curve (AUC) values of 1.00, indicating that these models perfectly discriminate between classes. According to the model estimated using the Xgboost algorithm, the most

important features are highlighted in Figure 3. Especially, these features include mismatching positions (21st position, 17th position, 6th position, 4th position, and 3rd position).

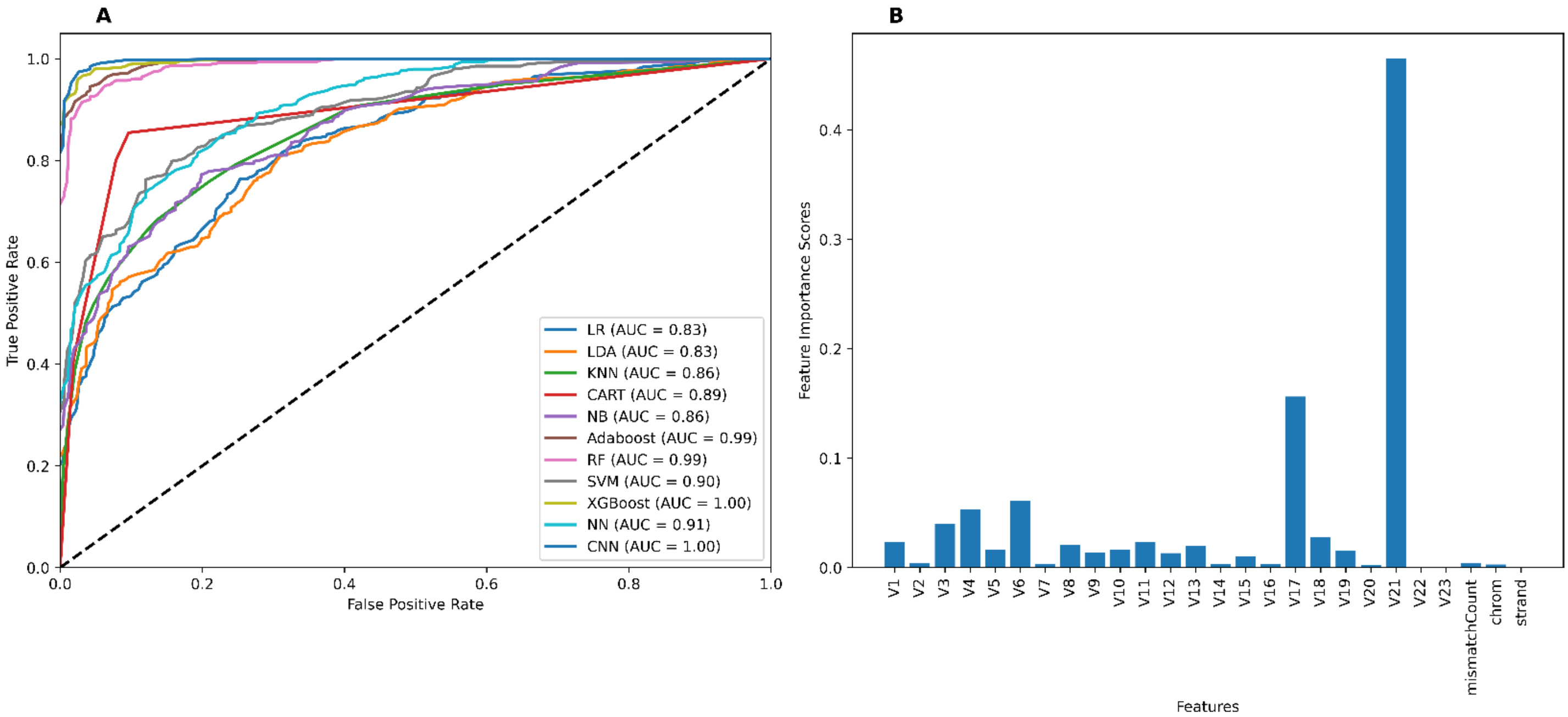

Figure 3. (A) Combined ROC Curve for Multiple Classifiers. **(B)** Feature importance for Xgboost algorithm

4. Discussion

In this study, the effect of Off-Target regions in the CRISPR Cas9 system is investigated using LCA in the TP53 gene. Before the analysis, the data was encoded in a format suitable for machine learning classifiers to better understand the mismatches between gRNA and target sequences. This encoding process identified potential mismatches in the 23-nucleotide long segment, including the PAM site within the gRNA sequence. Each mismatch case was handled separately using multi-categorical coding as the result of 16 possible scenarios. However, the matching regions between the gRNA and the target sequence were excluded from this coding, and the analysis focused solely on the mismatches. This coding process created 13 different categories of mismatches. Matching regions were coded as '1' and included in the analyses. A multi-categorical method was used for the general analyses. On the other hand, the data was processed using the one-hot encoding method for deep learning algorithms (NN and CNN), so the algorithms could analyze the mismatches more effectively. The CRISPR Cas9 system's effects were examined on the Off-Target regions in TP53 gene using both encoding approaches.

Latent class analysis was first used to determine the subclasses of Off-Target positions. Thus, the level of target sites was determined by the number and location of mismatches between the gRNA and target sequences. While machine learning models are generally estimated as Off/On-Target for CRISPR applications in the literature, this research estimated machine learning models across more than two Off-Target levels using latent class models based on Off-Target positions. After each latent class model was tested with the LMR test, models were selected as 3 latent classes using information criteria from the significant models. Then, machine learning algorithms were used to estimate the positions of the Off-Target levels. Off-Target positions were estimated using Logistic Regression, Linear Discriminant Analysis, K-Nearest Neighbors, Regression Trees, Naïve Bayes, Support Vector Machines, Xgboost, Adaboost, Random Forest, Neural Networks and Convolutional Neural Networks classifiers. It was found that the Xgboost algorithm with multi-categorical encoding achieved the highest accuracy in both the training and test datasets. According to the analysis results, Off-Target levels contribute positively to the increase in accuracy in neural networks (train: 100%, test: 98.9%) and convolutional neural network applications (train: 100%, test: 100%), which were implemented using the one-hot encoding method. Deep learning classifiers were supported by other performance ratios including precision, recall and F1 for both train and test dataset. Furthermore, deep networks, boosting algorithms, decision trees and support vector machines were notably discriminated from traditional classifiers such as logistic regression, linear discriminant analysis, K-near neighbors, and naïve Bayes. The model was also estimated using the Xgboost algorithm. Important features included mismatching positions (21st position, 17th position, 6th position, 4th position and 3rd position).

There are many studies on Off-Target prediction using both machine learning and deep learning in literature. The accuracies obtained using traditional machine learning algorithms in these studies are shown in Table 5.

Table 5. A summary of studies applying machine learning methods for off-target in CRISPR-Cas9 by[56]

Study	Models	Prediction metric/results
Vora et al. [9]	RF	ACC:0.970

S.A. Chen & Tran [57]	LR	ACC:0.940
Aktaş et al.[58]	NNET	ACC:0.960
Z.R. Zhang & Jiang [59]	SVM	ACC:0.982
Dhanjal et al. [30]	Xgboost	ACC: 0.914
Kose et al. [60]	Xgboost	ACC: 1.000
Trivedi et al. [29]	SVM	AUC: 0.980
Chuai et al. [61]	CNN	AUC: 0.804
Abadi et al. [62]	RF	AUC:0.960
Peng et al. [63]	RF	AUC:0.990
Lazzarotto et al. [64]	RF	AUC:0.995
Listgarten et al. [16]	Boosted Regression Trees	AUC:0.980
Doench & Root [18]	SVM+LR	AUC:0.800
S.Zhang et al. [65]	Adaboost	AUC:0.938

Machine learning and deep learning techniques hold great potential in genome editing studies, it provides high accuracy and classification rates in Off-Target prediction. Especially, algorithms demonstrate superior performance in both ACC and AUC metrics such as random forest, support vector machines and convolutional neural networks. Vora et al. [9] and Z.R. Zhang & Jiang [59] indicate significant progress in this field, they have found ACC of over 97%. In AUC metrics, Lazzarotto et al. [64] and Peng et al. [63] achieved success rates above 99%. Unlike Kose et al. [60], which examined classes in three regions (Intron, Intergenic and Exon), LCA and machine learning were applied solely to the exon region in this study. Moreover, a convolutional neural network model was incorporated into this study. This study demonstrates that LCA and machine learning applications performed only on exons give better results in determining Off-Target levels. It also shows that the one-hot encoding method provides more meaningful results in predicting outcomes using neural networks and convolutional neural network methods. In the future direction, we plan to hybridize our procedure with combined models.

5. Conclusion

Consequently, to minimize Off-Target effects, the gRNA must exactly match the PAM sequence. The PAM is recognized by the Cas protein and serves as a signal for binding and cutting the target sequence. Especially, the PAM sequence is required to initiate cutting at the target sequence in some CRISPR systems such as Cas9. If the suitable PAM is not found in the target sequence by the gRNA, Cas9 or a similar enzyme will not be able to cut the DNA, and genetic editing will not occur. Selecting suitable PAM sequence can reduce the risk of undesirable Off-Target effects and increase the accuracy of genetic editing processes. Therefore, addressing the right PAM sequence plays a crucial role in genetic editing studies using the CRISPR Cas system. gRNA allows the CRISPR Cas system to direct the target sequence. This allows genetic editing to occur directly at the desired site, as gRNA can be highly specific to the target sequence.Thus, unwanted Off-Target effects are reduced, increasing the precision of the genetic editing process. This also enables specific changes to be made in specific regions of certain genes. gRNA design offers a wide range of applications for editing various genes or targeted genetic regions. Therefore, gRNA is a crucial component in genetic editing studies using Cas systems. Carefully designed gRNAs ensure that the desired genetic edits are made, and targeted variants are achieved. These findings offer valuable insights into the complex interactions between CRISPR gRNA and genetic sequences. It has been suggested that a new strategy development is to address the challenges caused by genomic composition and the significant differences between the gRNA and DNA target regions. This study contributes to potential future research aimed at optimizing genome editing by customizing CRISPR Cas9 to target protospacer DNA.

Data availability statement

The datasets involved in this study are publicly available from the following links:

The raw data: https://www.ncbi.nlm.nih.gov/nuccore/NG_017013.2?from=5001&to=24149&report=fasta

Conflict of interests

The Authors have no conflicts of interest to disclose.

References

[1] G. Liang, H. Zhang, D. Lou, and D. Yu, “Selection of highly efficient sgRNAs for CRISPR/Cas9-based plant genome editing,” *Sci. Rep.*, vol. 6, pp. 1–8, 2016, doi: 10.1038/srep21451.

[2] H. Li, Y. Yang, W. Hong, M. Huang, M. Wu, and X. Zhao, “Applications of genome editing technology in the targeted therapy of human diseases: mechanisms, advances and prospects,” *Signal Transduct. Target. Ther.*, vol. 5, no. 1, 2020, doi: 10.1038/s41392-019-0089-y.

[3] J. H. Zhang, P. Adikaram, M. Pandey, A. Genis, and W. F. Simonds, “Optimization of genome editing through CRISPR-Cas9 engineering,” *Bioengineered*, vol. 7, no. 3, pp. 166–174, 2016, doi: 10.1080/21655979.2016.1189039.

[4] Y. Chen and Y. Zhang, “Application of the CRISPR/Cas9 System to Drug Resistance in Breast Cancer,” *Adv. Sci.*, vol. 5, no. 6, 2018, doi: 10.1002/advs.201700964.

[5] Y. Ishino, M. Krupovic, and P. Forterre, “History of CRISPR-Cas from Encounter with a Mysterious,” *J. Bacteriol.*, vol. 200, no. 7, pp. 1–17, 2018.

[6] J. C. Rose *et al.*, “Suppression of unwanted CRISPR-Cas9 editing by co-administration of catalytically inactivating truncated guide RNAs,” *Nat. Commun.*, vol. 11, no. 1, pp. 1–11, 2020, doi: 10.1038/s41467-020-16542-9.

[7] G. hui Chuai, Q. L. Wang, and Q. Liu, “In Silico Meets In Vivo: Towards Computational CRISPR-Based sgRNA Design,” *Trends Biotechnol.*, vol. 35, no. 1, pp. 12–21, 2017, doi: 10.1016/j.tibtech.2016.06.008.

[8] F. Jiang and J. A. Doudna, “CRISPR – Cas9 Structures and Mechanisms,” *Annu. Rev. Biophys.*, vol. 46, pp. 505–531, 2017.

[9] D. S. Vora, Y. Verma, and D. Sundar, “A Machine Learning Approach to Identify the Importance of Novel Features for CRISPR/Cas9 Activity Prediction,” *Biomolecules*, vol. 12, no. 8, pp. 1–15, 2022, doi: 10.3390/biom12081123.

[10] S. Riesenberg, N. Helmbrecht, P. Kanis, T. Maricic, and S. Pääbo, “Improved gRNA secondary structures allow editing of target sites resistant to CRISPR-Cas9 cleavage,” *Nat. Commun.*, vol. 13, no. 1, pp. 1–8, 2022, doi: 10.1038/s41467-022-28137-7.

[11] S. H. Kang *et al.*, “Prediction-based highly sensitive CRISPR off-target validation using target-specific DNA enrichment,” *Nat. Commun.*, vol. 11, no. 1, pp. 1–11, 2020, doi: 10.1038/s41467-020-17418-8.

[12] L. O. W. Wilson, A. R. O’Brien, and D. C. Bauer, “The current state and future of CRISPR-Cas9 gRNA design tools,” *Front. Pharmacol.*, vol. 9, no. JUN, pp. 1–6, 2018, doi: 10.3389/fphar.2018.00749.

[13] J. Zischewski, R. Fischer, and L. Bortesi, “Detection of on-target and off-target mutations generated by CRISPR/Cas9 and other sequence-specific nucleases,” *Biotechnol. Adv.*, vol. 35, no. 1, pp. 95–104, 2017, doi: 10.1016/j.biotechadv.2016.12.003.

[14] M. L. Leibowitz *et al.*, “Chromothripsis as an on-target consequence of CRISPR–Cas9 genome editing,” *Nat. Genet.*, vol. 53, no. 6, pp. 895–905, 2021, doi: 10.1038/s41588-021-00838-7.

[15] P. F. Kuan, S. Powers, S. He, K. Li, X. Zhao, and B. Huang, “A systematic evaluation of nucleotide properties for CRISPR sgRNA design,” *BMC Bioinformatics*, vol. 18, no. 1, pp. 1–9, 2017, doi: 10.1186/s12859-017-1697-6.

[16] J. Listgarten *et al.*, “Prediction of off-target activities for the end-to-end design of CRISPR guide RNAs,” *Nat. Biomed. Eng.*, vol. 2, no. 1, pp. 38–47, 2018, doi: 10.1038/s41551-017-0178-6.

[17] M. Haeussler *et al.*, “Evaluation of off-target and on-target scoring algorithms and integration into the guide RNA selection tool CRISPOR,” *Genome Biol.*, vol. 17, no. 1, pp. 1–12, 2016, doi: 10.1186/s13059-016-1012-2.

[18] J. G. Doench and D. . Root, “Optimized sgRNA design to maximize activity and minimize off-target effects of CRISPR-Cas9 Synthesis of an arrayed sgRNA library targeting the human genome,” *Nat. Biotechnol.*, vol. 34, no. 2, pp. 184–191, 2016, doi: 10.1038/nbt.3437.Optimized.

[19] P. D. Hsu *et al.*, “DNA targeting specificity of RNA-guided Cas9 nucleases,” *Nat. Biotechnol.*, vol. 31, no. 9, pp. 827–832, 2013, doi: 10.1038/nbt.2647.

[20] J.-P. Fortin, A. Lun, and L. Hoberecht, “On-target and off-target scoring for CRISPR gRNAs.” [Online]. Available: https://bioconductor.org/packages/devel/bioc/vignettes/crisprScore/inst/doc/crisprScore.html#1_Overview

[21] H. Peng, Y. Zheng, Z. Zhao, and J. Li, “Multigene editing: Current approaches and beyond,” *Brief. Bioinform.*, vol. 22, no. 5, pp. 1–11, 2021, doi: 10.1093/bib/bbaa396.

[22] H. Wang, M. Guo, H. Wei, and Y. Chen, “Targeting p53 pathways: mechanisms, structures, and advances in therapy,” *Signal Transduct. Target. Ther.*, vol. 8, no. 1, pp. 1–35, 2023, doi: 10.1038/s41392-023-01347-1.

[23] G. Cullot *et al.*, “Cell cycle arrest and p53 prevent ON-target megabase-scale rearrangements induced by CRISPR-Cas9,” *Nat. Commun.*, vol. 14, no. 1, pp. 1–16, 2023, doi: 10.1038/s41467-023-39632-w.

[24] S. Raschka and V. Mirjalili, *Python machine learning: Machine learning and deep learning with Python, scikit-learn, and TensorFlow 2*. Birmingham: Packt Publishing, 2019.

[25] B. Bin Jia and M. L. Zhang, “Multi-Dimensional Classification via Decomposed Label Encoding,” *IEEE Trans. Knowl. Data Eng.*, vol. 35, no. 2, pp. 1844–1856, 2023, doi: 10.1109/TKDE.2021.3100436.

[26] D. Liu, J. Zhao, J. Wu, G. Yang, and F. Lv, “Multi-category classification with label noise by robust binary loss,” *Neurocomputing*, vol. 482, pp. 14–26, 2022, doi: 10.1016/j.neucom.2022.01.031.

[27] L. Yu, R. Zhou, R. Chen, and K. K. Lai, “Missing Data Preprocessing in Credit Classification: One-Hot Encoding or Imputation?,” *Emerg. Mark. Financ. Trade*, vol. 58, no. 2, pp. 472–482, 2022, doi: 10.1080/1540496X.2020.1825935.

[28] H. Lu, “Quasi-orthonormal Encoding for Machine Learning Applications,” *Proc. 19th Python Sci. Conf.*, pp. 11–17, 2020, doi: 10.25080/majora-342d178e-002.

[29] T. B. Trivedi *et al.*, “Crispr2vec: Machine Learning Model Predicts Off-Target Cuts of CRISPR systems,” *bioRxiv*, pp. 1–19, 2020, [Online]. Available: https://doi.org/10.1101/2020.10.28.359885

[30] J. K. Dhanjal, S. Dammalapati, S. Pal, and D. Sundar, “Evaluation of off-targets predicted by sgRNA design tools,” *Genomics*, vol. 112, no. 5, pp. 3609–3614, 2020, doi: 10.1016/j.ygeno.2020.04.024.

[31] Aurélien Géron, *Hands-on machine learning with Scikit-Learn, Keras and TensorFlow: concepts, tools, and techniques to build intelligent systems*. Sebastopol: O’REILLY, 2019. [Online]. Available: https://www.oreilly.com/library/view/hands-on-machine-learning/9781492032632/

[32] H. Chung, "Latent class models," in *Encyclopedia of Epidemiology*, 1th ed., S. Boslaugh and L.-A. McNutt, Eds., California: SAGE Publications, Inc. All, 2008, pp. 584–586.

[33] Y. Lo, N. R. Mendel, and D. B. Rubin, "Testing the number of components in a normal mixture," *Biometrika*, vol. 88, no. 3, pp. 767–778, 2001, doi: https://doi.org/10.1093/biomet/88.3.767.

[34] Q. H. Vuong, "Likelihood Ratio Tests for Model Selection and Non-Nested Hypotheses," *Econometrica*, vol. 57, no. 2, pp. 307–333, 1989.

[35] Q. Chen, W. Luo, G. J. Palardy, R. Glaman, and A. McEnturff, "The Efficacy of Common Fit Indices for Enumerating Classes in Growth Mixture Models When Nested Data Structure Is Ignored: A Monte Carlo Study," *SAGE Open*, vol. 7, no. 1, pp. 1–19, 2017, doi: 10.1177/2158244017700459.

[36] H. Akaike, "Information Theory and an Extension of the Maximum Likelihood Principle.," in *International Symposium on Information Theory*, F. Petrov, B.N. and Csaki, Ed., Budapest, 1973, pp. 267–281.

[37] G. Schwarz, "Estimating the Dimension of a Model," *Ann. Stat.*, vol. 6, no. 2, pp. 461–464, 1978.

[38] H. Bozdogan, "Model selection and Akaike's Information Criterion (AIC): The general theory and its analytical extensions," *Psychometrika*, vol. 52, no. 3, pp. 345–370, 1987, doi: 10.1007/BF02294361.

[39] H. D.-G. Acquah, "Weighted Average Information Criterion for Selection of an Asymmetric Price Relationship," *Alanya Akad. Bakış*, vol. 2, no. 2, pp. 147–155, 2018, doi: 10.29023/alanyaakademik.343737.

[40] H. Bozdogan, "Akaike's information criterion and recent developments in information complexity," *J. Math. Psychol.*, vol. 44, no. 1, pp. 62–91, 2000, doi: 10.1006/jmps.1999.1277.

[41] K. L. Nylund *et al.*, "Deciding on the number of classes in latent class analysis and growth mixture modeling : a monte carlo simulation study," *Struct. Equ. Model.*, vol. 14, no. 4, pp. 535–569, 2007.

[42] T. Asparouhov and B. Muthén, "Auxiliary Variables in Mixture Modeling: Three-Step Approaches Using Mplus," *Struct. Equ. Model.*, vol. 21, no. 3, pp. 329–341, 2014, doi: 10.1080/10705511.2014.915181.

[43] M. Qiu, “A Tutorial on Bayesian Latent Class Analysis Using JAGS,” *J. Behav. Data Sci.*, vol. 2, no. 2, pp. 127–155, Dec. 2022, doi: 10.35566/jbds/v2n2/qiu.

[44] J.-Y. Tein, S. Coxe, and H. Cham, “Statistical Power to Detect the Correct Number of Classes in Latent Profile Analysis,” *Struct. Equ. Model.*, vol. 20, no. 4, pp. 640–657, 2013, doi: 10.1080/10705511.2013.824781.

[45] E. A. Granado, “Comparing Three Effect Sizes for Latent Class Analysis,” University of North Texas, 2015. [Online]. Available: https://digital.library.unt.edu/ark:/67531/metadc822835/m2/1/high_res_d/dissertation.pdf

[46] K. P. Murphy, *Machine Learning A Probabilistic Perspective*. London: The MIT Press, 2012. doi: 10.1007/978-94-011-3532-0_2.

[47] E. Alpaydın, *Introduction to Machine Learning*, Second. Massachusetts: The MIT Press, 2010.

[48] B. Efron and T. Hastie, *Computer age statistical inference: Algorithms, evidence, and data science*. 2016. doi: 10.1017/CBO9781316576533.

[49] M. Mohammed, M. B. Khan, and E. B. M. Bashier, *Machine Learning Algorithms and Applications*. Boca Raton, FL: Taylor & Francis, 2017.

[50] X. Vasques, *Machine Learning Theory and Applications Hands-on Use Cases with Python*. New Jersey: Wiley & Sons Inc., 2024.

[51] C. Bentéjac, A. Csörgő, and G. Martínez-Muñoz, *A comparative analysis of gradient boosting algorithms*, vol. 54, no. 3. Springer Netherlands, 2021. doi: 10.1007/s10462-020-09896-5.

[52] C. M. Bishop, *Pattern Recognition and Machine Learning*. Singapore: Springer, 2006. doi: 10.1007/BF00746534.

[53] A. Ozcift and A. Gulten, “Classifier ensemble construction with rotation forest to improve medical diagnosis performance of machine learning algorithms,” *Comput. Methods Programs Biomed.*, vol. 104, no. 3, pp. 443–451, 2011, doi: 10.1016/j.cmpb.2011.03.018.

[54] A. Bailly *et al.*, “Computer Methods and Programs in Biomedicine Effects of dataset size and

interactions on the prediction performance of logistic regression and deep learning models," *Comput. Methods Programs Biomed.*, vol. 213, p. 106504, 2022, doi: 10.1016/j.cmpb.2021.106504.

[55] Q. Cao, H. Sun, H. Wang, X. Liu, Y. Lu, and L. Huo, "Comparative study of neonatal brain injury fetuses using machine learning methods for perinatal data," *Comput. Methods Programs Biomed.*, vol. 240, pp. 1–7, 2023, doi: 10.1016/j.cmpb.2023.107701.

[56] Z. Sherkatghanad, M. Abdar, J. Charlier, and V. Makarenkov, "Using traditional machine learning and deep learning methods for on- and off-target prediction in CRISPR/Cas9: a review," *Brief. Bioinform.*, vol. 24, no. 3, pp. 1–25, 2023, doi: 10.1093/bib/bbad131.

[57] S. A. Chen and E. Tran, "Optimizing Precision Genome Editing through Machine Learning," 2018.

[58] Ö. Aktaş, E. Doğan, and T. Ensari, "Derin Öğrenmeyle CRISPR/CAS9 Hedefleme Tahmini," in *2019 Scientific Meeting on Electrical-Electronics & Biomedical Engineering and Computer Science (EBBT 2019)*, İstanbul, 2019, p. 277 (1 Vol).

[59] Z. R. Zhang and Z. R. Jiang, "Effective use of sequence information to predict CRISPR-Cas9 off-target," *Comput. Struct. Biotechnol. J.*, vol. 20, pp. 650–661, 2022, doi: 10.1016/j.csbj.2022.01.006.

[60] A. M. Kose, O. Kocadagli, C. Tas, C. Aktan, and O. Mert, "Unveiling Off-Target Mutations in CRISPR Guide RNAs : Implications for Gene Region Specificity," *Cris. J.*, vol. 7, no. 3, pp. 168–178, 2024, doi: 10.1089/crispr.2024.0002.

[61] G. Chuai *et al.*, "DeepCRISPR: Optimized CRISPR guide RNA design by deep learning," *Genome Biol.*, vol. 19, no. 1, pp. 1–18, 2018, doi: 10.1186/s13059-018-1459-4.

[62] S. Abadi, W. X. Yan, D. Amar, and I. Mayrose, "A machine learning approach for predicting CRISPR-Cas9 cleavage efficiencies and patterns underlying its mechanism of action," *PLoS Comput. Biol.*, vol. 13, no. 10, pp. 1–24, 2017, doi: 10.1371/journal.pcbi.1005807.

[63] H. Peng, Y. Zheng, Z. Zhao, T. Liu, and J. Li, "Recognition of CRISPR/Cas9 off-target sites through ensemble learning of uneven mismatch distributions," *Bioinformatics*, vol. 34, no. 17, pp. i757–i765, 2018, doi: 10.1093/bioinformatics/bty558.

[64] C. R. Lazzarotto *et al.*, “CHANGE-seq reveals genetic and epigenetic effects on CRISPR–Cas9 genome-wide activity,” *Nat. Biotechnol.*, vol. 38, no. 11, pp. 1317–1327, 2020, doi: 10.1038/s41587-020-0555-7.

[65] S. Zhang, X. Li, Q. Lin, and K. C. Wong, “Synergizing CRISPR/Cas9 off-target predictions for ensemble insights and practical applications,” *Bioinformatics*, vol. 35, no. 7, pp. 1108–1115, 2019, doi: 10.1093/bioinformatics/bty748.

TARGET	T	A	C	T	G	A	T	A	G	T	T	T	C	T	G	G	G	T	G	A	A	G	G
NUCLEOTIDE POSITION	1	2	3	4	5	6	7	8	9	10	11	12	13	14	15	16	17	18	19	20	21	22	23
OFF-TARGET	T	A	G	T	G	A	T	A	G	T	T	T	C	T	G	G	G	T	G	A	A	G	G
MATCHING-X																							
A_X	0	1	0	0	0	1	0	1	0	0	0	0	0	0	0	0	0	0	0	1	1	0	0
T_X	1	0	0	1	0	0	1	0	0	1	1	1	0	1	0	0	0	1	0	0	0	0	0
G_X	0	0	1	0	1	0	0	0	1	0	0	0	0	0	1	1	1	0	1	0	0	1	1
C_X	0	0	0	0	0	0	0	0	0	0	0	0	1	0	0	0	0	0	0	0	0	0	0
NO CHANGE	1	1	0	1	1	1	1	1	1	1	1	1	1	1	1	1	1	1	1	1	1	1	1
TRANSITION	0	0	1	0	0	0	0	0	0	0	0	0	0	0	0	0	0	0	0	0	0	0	0

Figure 1. One-hot encoding for DNA sequence adopted from [30]

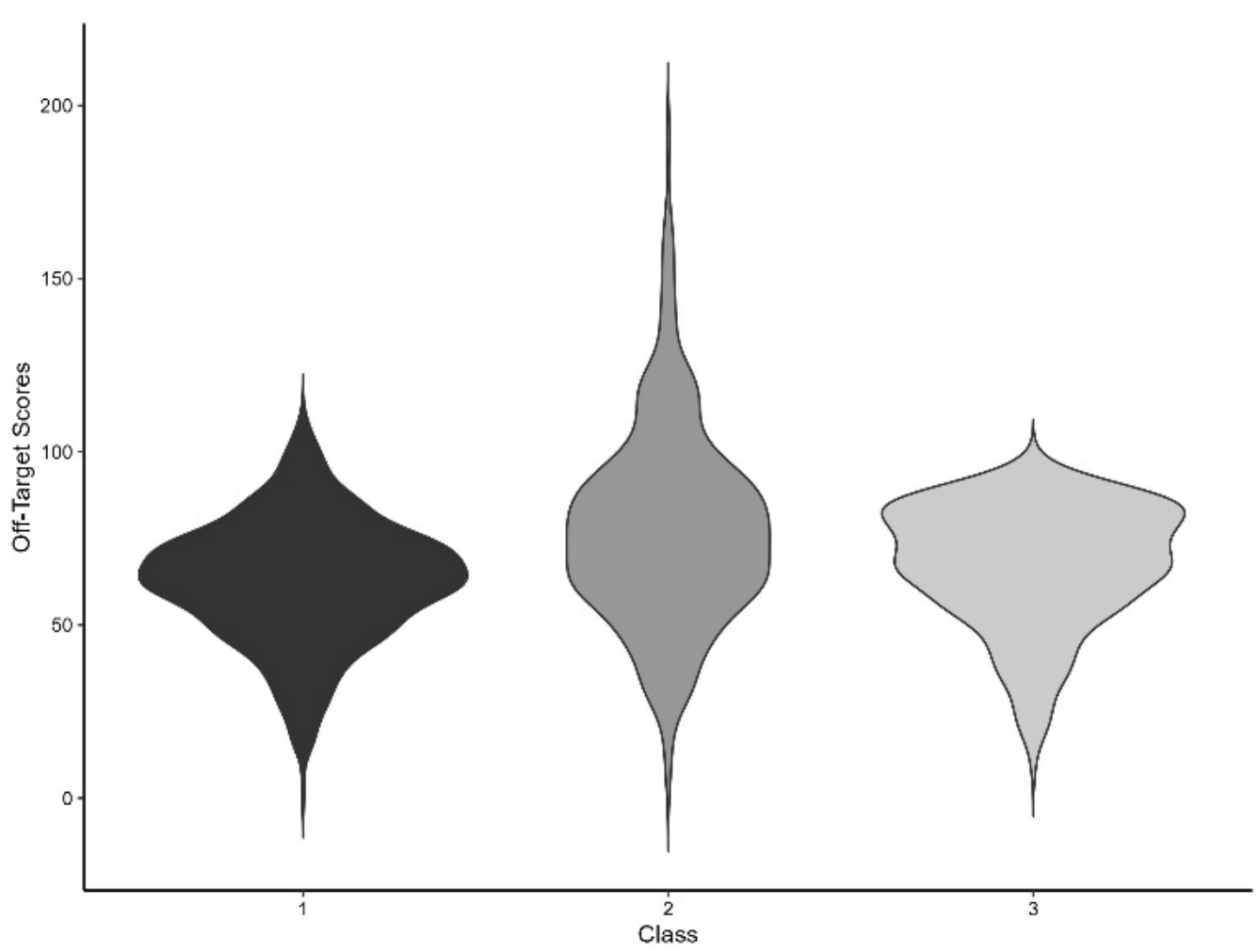

Figure 2. Off-Target scores for each class.

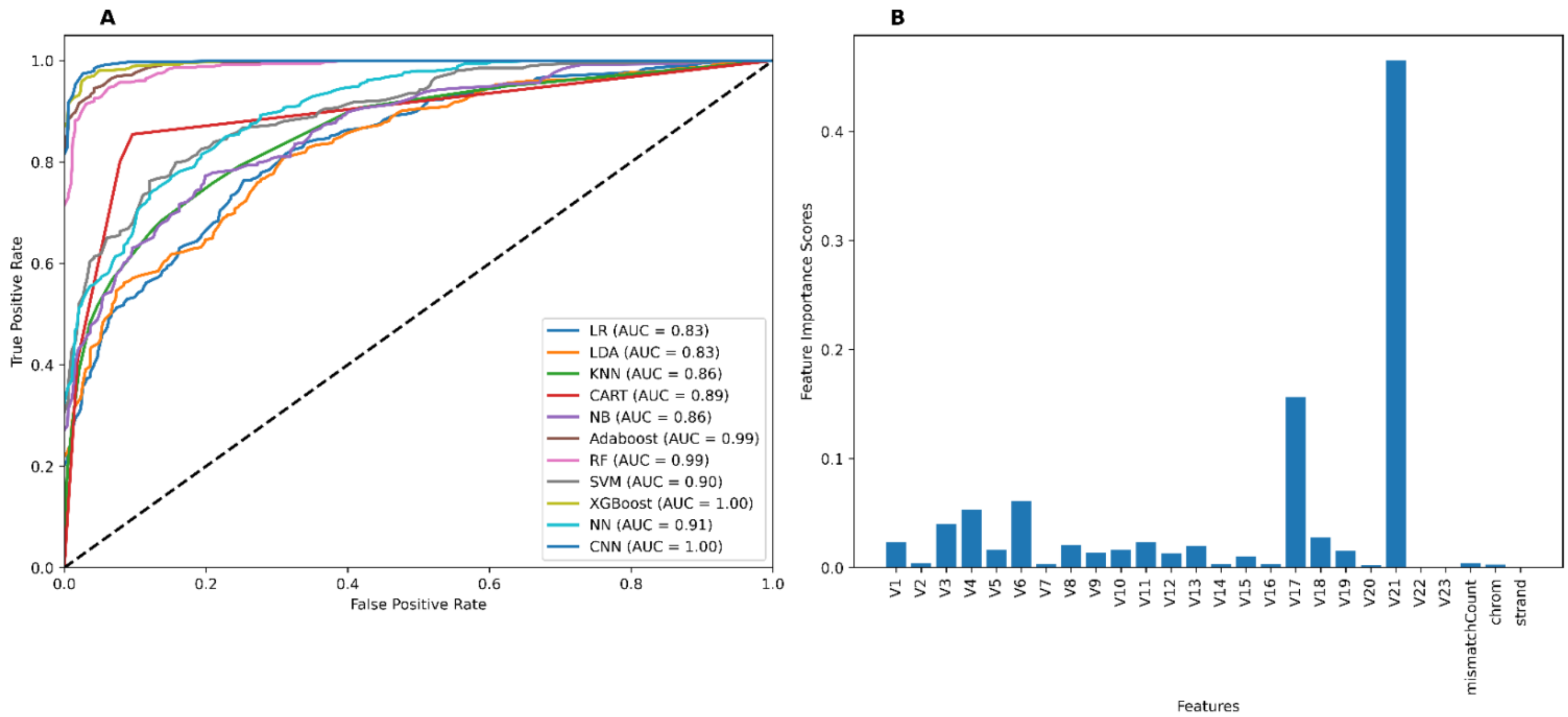

Figure 3. (A) Combined ROC Curve for Multiple Classifiers. **(B)** Feature importance for Xgboost algorithm

Table 1. Multi-categorical coding

gRNA	Target Sequence	Matching
A	A	1
C	A	2
G	A	3
T	A	4
C	C	1
A	C	5
G	C	6
T	C	7
G	G	1
A	G	8
C	G	9
T	G	10
T	T	1
A	T	11
C	T	12
G	T	13

Table 2. Descriptive characteristics of positions

Variables		Frequency (n=947)	Percent (%)	Variables		Frequency (n=947)	Percent (%)
V1	match-1	682	72	V14	match-1	756	79.8
	C-A/A-C	65	6.9		C-A/A-C	84	8.9
	G-A/A-G	28	2.9		G-A/A-G	15	1.5
	T-A/A-T	41	4.4		T-A/A-T	39	4.2
	G-C/C-G	39	4.1		G-C/C-G	10	1
	T-C/C-T	45	4.8		T-C/C-T	36	3.8
	T-G/G-T	47	4.9		T-G	7	0.7
V2	match-1	740	78.1	V15	match-1	750	79.2
	C-A/A-C	17	1.8		C-A/A-C	84	8.8
	G-A/A-G	33	3.4		G-A	15	1.6
	T-A/A-T	66	6.9		T-A/A-T	21	2.2
	G-C/C-G	40	4.2		G-C	32	3.4
	T-C/C-T	17	1.8		T-C/C-T	40	4.2

	T-G/G-T	34	3.4		G-T	5	0.5
V3	match-1	788	83.2	V16	match-1	675	71.3
	C-A/A-C	31	3.3		C-A/A-C	54	5.7
	G-A/A-G	16	1.6		G-A/A-G	29	3.1
	T-A/A-T	31	3.3		T-A/A-T	92	9.7
	G-C/C-G	30	3.2		G-C/C-G	23	2.5
	T-C/C-T	20	2.1		T-C/C-T	18	1.9
	T-G/G-T	31	3.2		T-G/G-T	56	5.9
V4	match-1	777	82	V17	match-1	631	66.6
	C-A	6	0.6		C-A/A-C	32	3.4
	G-A/A-G	35	3.7		G-A/A-G	38	4.1
	T-A/A-T	29	3.1		T-A/A-T	45	4.7
	C-G	20	2.1		G-C/C-G	24	2.6
	C-T	25	2.6		T-C/C-T	103	10.9
	T-G/G-T	55	5.8		T-G/G-T	74	7.8
V5	match-1	750	79.2	V18	match-1	690	72.9
	C-A/A-C	41	4.4		A-C	24	2.5
	G-A/A-G	70	7.4		/A-G	35	3.7
	T-A	15	1.6		A-T	19	2
	G-C/C-G	46	4.9		G-C/C-G	36	3.8
	T-C	13	1.4		T-C/C-T	93	9.8
	T-G	12	1.3		T-G/G-T	50	5.3
V6	match-1	807	85.2	V19	match-1	689	72.8
	C-A/A-C	17	1.8		C-A/A-C	62	6.6
	G-A/A-G	29	3		G-A/A-G	20	2.2
	T-A/A-T	34	3.6		T-A/A-T	42	4.4
	G-C/C-G	12	1.2		G-C/C-G	65	6.9
	T-C/C-T	41	4.3		T-C/C-T	42	4.5
	T-G	7	0.7		T-G/G-T	27	2.8
V7	match-1	718	75.8	V20	match-1	741	78.2
	C-A	24	2.5		C-A/A-C	44	4.6
	G-A/A-G	93	9.8		G-A/A-G	19	2
	T-A	61	6.4		T-A/A-T	62	6.5
	C-G	9	1		G-C/C-G	13	1.4
	T-G	42	4.4		T-C/C-T	27	2.8
					T-G/G-T	41	4.4
V8	match-1	592	62.5	V21	match-1	255	26.9
	A-C	33	3.5		C-A/A-C	113	12
	A-G	125	13.2		G-A/A-G	129	13.6
	G-C/C-G	99	10.5		T-A/A-T	144	15.2
	T-C	39	4.1		G-C/C-G	83	8.8
	T-G	59	6.2		T-C/C-T	106	11.2
					T-G/G-T	117	12.3
V9	match-1	707	74.7	V22	match-1	454	47.9
	C-A/A-C	6	1.1		A-G	493	52.1
	G-A/A-G	50	5.3	V23	match-1	947	100
	T-A/A-T	47	4.9	mismatchCount	0	1	0.1
	G-C/C-G	44	4.6		1	23	2.4
	T-C/C-T	51	5.3		2	268	28.3
	T-G/G-T	42	4.4		3	655	69.2

V10	match-1	748	79	Strand	+	490	51.7
	C-A/A-C	18	1.9		-	457	48.3
	G-A/A-G	31	3.2	class	1	401	42.3
	T-A/A-T	36	3.8		2	253	26.7
	G-C/C-G	35	3.7				
	T-C/C-T	59	6.2		3	293	30.9
	T-G/G-T	20	2.2				
V11	match-1	662	69.9	chrom	chrom1	75	7.9
	C-A/A-C	131	13.9		chrom2	79	8.3
	G-A/A-G	27	2.8		chrom3	58	6.1
	T-A/A-T	30	3.1		chrom4	56	5.9
	G-C/C-G	31	3.2		chrom5	51	5.4
	T-C/C-T	43	4.5		chrom6	49	5.2
	T-G/G-T	23	2.5		chrom7	46	4.9
V12	match-1	649	68.5		chrom8	45	4.8
	C-A/A-C	67	7.1		chrom9	37	3.9
	G-A/A-G	80	8.4		chrom10	50	5.3
	T-A/A-T	19	2		chrom11	52	5.5
	G-C/C-G	56	5.9		chrom12	42	4.4
	T-C/C-T	37	3.9		chrom13	35	3.7
	T-G/G-T	39	4.2		chrom14	29	3.1
V13	match-1	748	79		chrom15	20	2.1
	C-A/A-C	72	7.6		chrom16	38	4
	G-A/A-G	36	3.8		chrom17	31	3.3
	T-A	30	3.2		chrom18	17	1.8
	G-C/C-G	12	1.3		chrom19	26	2.7
	T-C	47	5		chrom20	27	2.9
					chrom21	13	1.4
	T-G	2	0.2		chrom22	25	2.6
					chrom23	42	4.4
					chromX	4	0.4

Table 3. Results of latent class analysis for multi-categorical observed variables

Class	Parameter	Log Likelihood	AIC	BIC	CAIC	LMR Test	Entropy

**2 class**	449	-21753.8	44405.6	46584.7	47033.7	<0.001	0.953
**3 class**	674	-20798.8	42945.6	**46216.7**	**46880.7**	**<0.001**	**0.981**
**4 class**	899	-20330.9	42459.7	46822.8	47721.8	<0.001	0.977
**5 class**	1124	-19815.4	41878.8	47333.9	48457.9	<0.001	0.977
**6 class**	1349	-19170	41037.9	47585.0	48934.1	<0.001	0.989

Table 4. The Machine learning classifiers results

	**Train (70%)**				**Test (30%)**			
**Model (10-fold cross validation)**	**ACC**	**Precision**	**Recall**	**F1 score**	**ACC**	**Precision**	**Recall**	**F1 Score**
Logistic regression (LR)	0.760	0.760	0.760	0.760	0.663	0.664	0.663	0.663
Linear discriminant analysis (LDA)	0.754	0.754	0.754	0.753	0.667	0.669	0.667	0.666
K-near neighbors (KNN)	0.766	0.774	0.766	0.763	0.604	0.645	0.604	0.592
Classification regression tree (CART)	0.872	0.861	0.861	0.861	0.811	0.824	0.821	0.821
Naïve Bayes (NB)	0.701	0.711	0.701	0.701	0.663	0.670	0.663	0.661
Support vector machine (SVM)	0.917	0.920	0.916	0.916	0.772	0.785	0.771	0.770
Xgboost	1.000	1.000	1.000	1.000	0.951	0.955	0.954	0.954
Adaboost	0.784	0.789	0.784	0.786	0.744	0.749	0.744	0.746

Random forest (RF)	0.947	0.934	0.932	0.932	0.854	0.860	0.853	0.854
Neural network (NN)	1.000	1.000	1.000	1.000	0.989	0.980	0.979	0.979
**Convolutional neural network (CNN)**	**1.000**	**1.000**	**1.000**	**1.000**	**1.000**	**1.000**	**1.000**	**1.000**

Table 5. A summary of studies applying machine learning methods for off-target in CRISPR-Cas9 by[56]

**Study**	**Models**	**Prediction metric/results**
Vora et al. [9]	RF	ACC:0.970
S.A. Chen & Tran [57]	LR	ACC:0.940
Aktaş et al.[58]	NNET	ACC:0.960
Z.R. Zhang & Jiang [59]	SVM	ACC:0.982
Dhanjal et al. [30]	Xgboost	ACC: 0.914
Kose et al. [60]	Xgboost	ACC: 1.000
Trivedi et al. [29]	SVM	AUC: 0.980
Chuai et al. [61]	CNN	AUC: 0.804
Abadi et al. [62]	RF	AUC:0.960
Peng et al. [63]	RF	AUC:0.990
Lazzarotto et al. [64]	RF	AUC:0.995
Listgarten et al. [16]	Boosted Regression Trees	AUC:0.980
Doench & Root [18]	SVM+LR	AUC:0.800
S.Zhang et al. [65]	Adaboost	AUC:0.938

Highlights

- Genome editing with CRISPR-Cas9
- Feature encoding system based for Off-Target positions in TP53 gene region
- Off-Target level Identification using Latent Class Analysis
- Off-Target effect evaluation using Machine Learning.
- Ensuring safety and efficacy in CRISPR-based Treatments

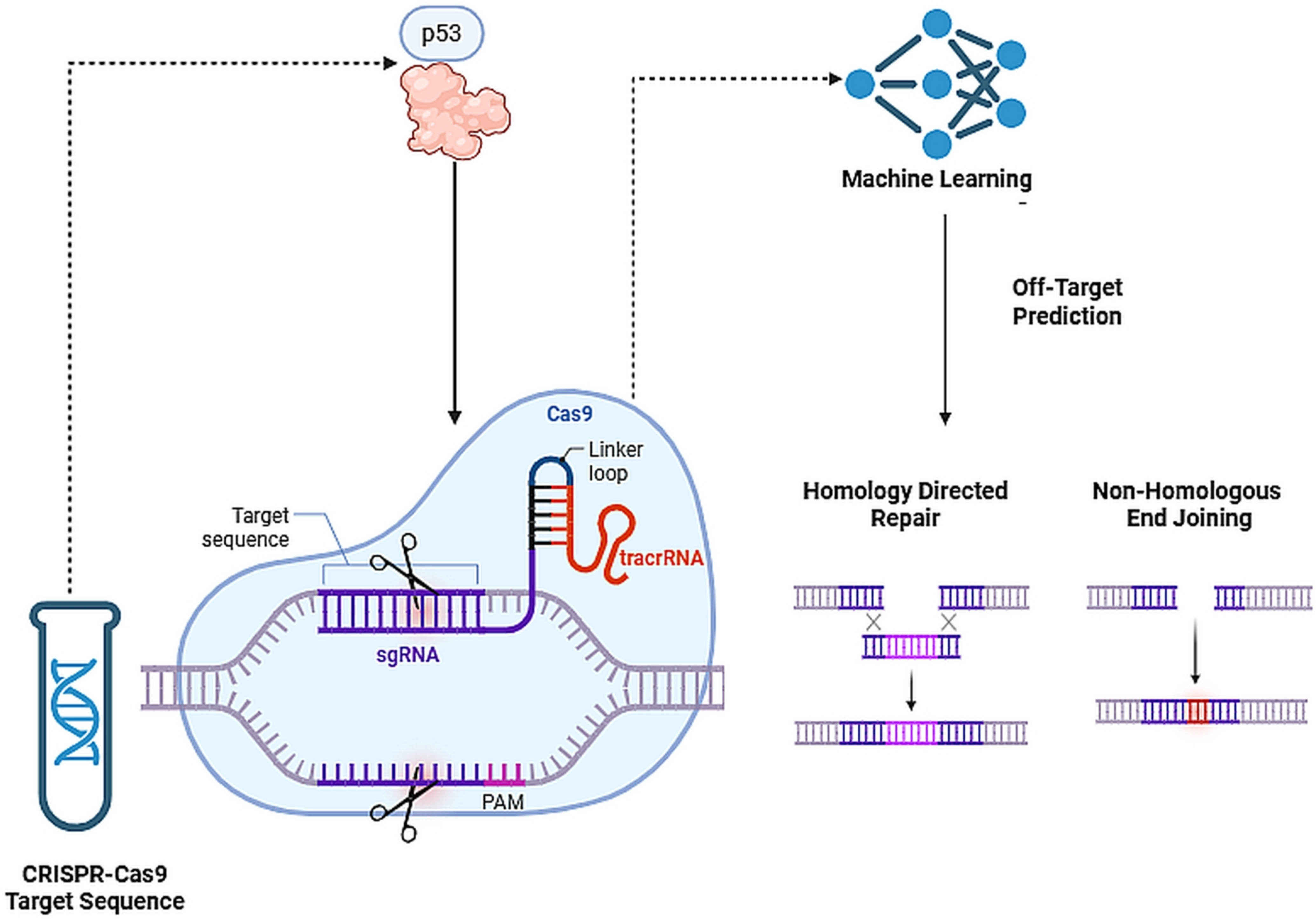
p53
Machine Learning
Off-Target
Prediction
Cas9
Linker
loop
Target
sequence
tracrRNA
sgRNA
PAM
Homology Directed
Repair
Non-Homologous
End Joining
CRISPR-Cas9
Target Sequence
